# PCNA in Pan-Cancer: A Prognostic Biomarker Unveiled Through a Data-Driven, Multidimensional Analysis of Transcriptomics, Immunity, and Functional Profiling

**DOI:** 10.1021/acsomega.5c03331

**Published:** 2025-06-26

**Authors:** Depanshi Pandit, Ravindranath Bilachi Sanganabasappa, Amardeep Dhillon, Sanjiban Chakrabarty

**Affiliations:** † Department of Biotechnology, Manipal Institute of Technology, Manipal, 76793 Manipal Academy of Higher Education, Manipal, Karnataka 576104, India-; ‡ The Institute for Mental and Physical Health and Clinical Translation, School of Medicine, 98475 Deakin University, Geelong, Waurn Ponds, Victoria 3216, Australia; § Department of Public Health Genomics, Manipal School of Life Sciences, Manipal Academy of Higher Education, Manipal 576104, India

## Abstract

Proliferating cell nuclear antigen (PCNA) is a central regulator of numerous cellular processes impacting DNA metabolism and genetic integrity, including DNA damage, cell cycle progression, and transcriptional regulation. PCNA aberrations manifest in different disease phenotypes, including neoplastic proliferation, chromatin disassembly, genomic instability, and impaired DNA repair. Although the role of PCNA in control of DNA homeostasis has been extensively studied in some cancers, its pan-cancer relevance in tumorigenesis, immune interactions, and therapeutic resistance remains underexplored. Here, we undertook a comprehensive analysis of publicly available databases to assess the relationship between PCNA expression and the immunological, survival, genetic, functional, and drug sensitivity profiles across multiple cancer types. PCNA mRNA levels were altered in across cancers and associated with altered cancer signaling networks constituting Wnt, Hippo, and mTOR pathways. Increased transcript levels were associated with poor overall survival in some cancers, including ACC, CESC, LGG, and THYM. Amplification was the predominant genetic alteration of PCNA in multiple tumors. In several tumors, upregulation of PCNA was linked with differences in tumor-infiltrating lymphocytes, and specific immune-inhibitors, and chemokines. Moreover, elevated PCNA expression was linked with increased sensitivity to several drugs, particularly to Navitoclax, NPK76-II-72-1, and Ciclopirox across cancers. Our study orients the first comprehensive pan-cancer analysis of PCNA, uncovering its prognostic significance and altered expression across various cancers through multiomics data. Unlike previous studies, tumor-specific genetic alterations, such as amplification and hypomethylation, and the paradoxical immune microenvironment linked to PCNA were explored, suggesting potential immune evasion mechanisms. Additionally, new therapeutic avenues reveal PCNA’s relationship with drug sensitivity to agents like Navitoclax and Ciclopirox, providing invaluable insights for pharmacological interventions.

## Graphical abstract

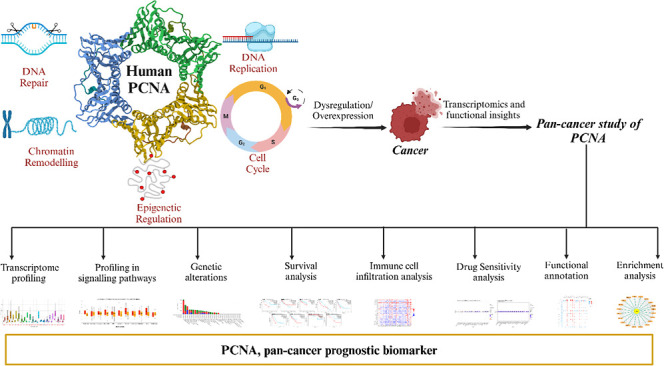

## 1 Introduction

Cancer encompasses a spectrum of disease phenotypes characterized by genetic variability and diverse mechanisms orchestrating tumor development, progression, and therapy resistance. The heterogeneity of cancers poses a major roadblock in the development of universal biomarkers and treatment methods. Thus, identifying therapeutic targets critical across multiple cancer types could transform early diagnosis and treatment paradigms, providing unified approach to combat this multifaceted disease.

Proliferating cell nuclear antigen (PCNA) is regarded as “the maestro of the replication fork”^1^ because it regulates DNA synthesis and directs repair components to damage sites within the replication fork. PCNA is a homotrimer with a toroidal structure that encircles DNA as a sliding clamp without dissociating, displaying quasi 6-fold symmetry which is critical for DNA replication and repair.^2–4^ It serves as a key scaffold in DNA replication and repair and assures the processivity factor of DNA polymerase δ during replication.^5^ PCNA and its myriad of binding partners participate in DNA synthesis, Okazaki fragment maturation, replication-coupled nucleosome assembly, chromatin assembly, DNA repair including nucleotide excision repair, mismatch repair, and translesion synthesis, DNA damage avoidance, apoptosis, sister chromatid cohesion, cell cycle, S-phase specific proteolysis, gene transcription, epigenomic mechanisms, glycolysis, NK cell activation, adipogenesis, and diet-induced obesity,^4,6–15^ by interacting with different proteins via its conserved PCNA-interacting protein box (PIP-Box) motif^1,16,17^ or by undergoing various post-translational modifications including phosphorylation, acetylation, methylation, ubiquitination and sumoylation.^1,2^ Eventually, occupying an enormous part in maintaining genomic equilibrium, which, when perturbed, may accelerate cancer development and progression.

PCNA expression is altered in a wide range of diseases, including reproductive conditions, viral infections, autoimmune disorders, and neurological disorders.^18–21^ Furthermore, it is a commonly used biomarker of cell proliferative activity, which is characteristic of aggressive cancers regardless of subtype.^4,22–34^ Because cell proliferation is required for cancer progression and PCNA is essential for DNA replication, it represents a promising target for anticancer therapies.^4^ However, despite its involvement in various signaling pathways and overexpression in some malignancies, the association between PCNA and tumor phenotypes across the pan-cancer spectrum remains unclear. Understanding this relationship is pivotal to establish the importance of PCNA as a novel universal biomarker for early cancer detection, therapeutic targeting, and patient stratification for personalized treatment approaches, particularly for aggressive cancers and in which patients that become unresponsive to traditional therapies.

The present study expounds a novel pan-cancer examination of PCNA employing The Cancer Genome Atlas (TCGA), Gene Expression Omnibus (GEO), Human Protein Atlas (HPA), and Genotype Tissue Expression (GTEx) databases. Our exhaustive investigation uncovered distinct PCNA expression patterns across multiple cancer types, with significant overexpression observed in the bladder, colon, esophagus, lung squamous cell, rectum, stomach, and testis. Genetic alterations, including amplification, mutation, and deep deletion, were associated with altered survival outcomes, particularly in adrenocortical carcinoma, nonsmall cell lung cancer, sarcoma, and cervical cancer. Notably, gene and functional enrichment analysis identified key PCNA-associated pathways such as error-prone translesion synthesis, DNA replication, DNA damage, DNA repair processes, and invasion, underscoring its role in tumorigenesis. Additionally, PCNA expression was significantly correlated with activated CD4+T cells, CD8+T cells, IDO1, and PDCD1, suggesting its potential role in modulating an altered tumor microenvironment and immune evasion mechanisms. Our analysis reveals a strong correlation between PCNA expression and drug sensitivity in top 30 drugs such as Navitoclax and Ciclopirox, indicating its crucial role in predicting therapeutic responses across multiple cancer types. These findings establish PCNA as a promising prognostic biomarker and highlight its potential for targeted therapeutic strategies in personalized and combinatorial cancer treatments.

## 2 Material and Methods

### 2.1 Expression Profiling

The TNMplot webpage (https://tnmplot.com/analysis/) was employed to analyze the differential expression of PCNA in various tumor tissues and adjacent normal tissues, utilizing RNA-seq data from 56,938 samples collected from the TCGA, Gene Expression Omnibus of the National Center for Biotechnology Information (NCBI-GEO), Therapeutically Applicable Research to Generate Effective Treatments (TARGET), and GTEx repositories.^35^ The RNA-seq quantification for TNMplot.com is count-based (HTSeq-counts) and normalized using DESeq2, followed by a second normalization layer to enable cross-data set comparability. Expression levels >10 were considered significant as per the database.^35^

The HPA (https://www.proteinatlas.org/) integrates various omics technologies, including antibody-based imaging, mass spectrometry-based proteomics, transcriptomics, and systems biology to map all human proteins in cells, tissues, and organs.^36^ PCNA mRNA expression in cell lines was explored using HPA’s cell line resource, which provides data on genome-wide RNA expression patterns for 1206 human cell lines, including 1132 cancer cell lines using RNA-seq.^36^

### 2.2 Mapping Expression in Cancer Pathways

PCNA levels were assessed across essential pathways such as WNT, MYC, and mTOR signaling to identify differential expression patterns in cancer types. The investigation was performed using the University of Alabama at Birmingham Cancer data analysis Portal (UALCAN) (https://ualcan.path.uab.edu/index.html), which derives the protein expression data from the CPTAC (Clinical Proteomic Tumor Analysis Consortium) and the International Cancer Proteogenome Consortium (ICPC) registries.^37,38^ The analysis considered pathway-level somatic changes such as mutations, copy number alterations (CNAs), whole exome, and proteomic profiles in normal, altered and other samples. Only “altered pathway status” and “others” sample categories were taken into consideration for graphical representation using R software.^39^

### 2.3 Genetic Alteration Analysis

The cBioPortal (http://www.cbioportal.org) is an interactive platform for analyzing multimodal cancer genomics data, such as somatic mutations, DNA copy number changes (CNAs), and DNA methylation.^40^ The alterations in PCNA among TCGA tumor types were investigated by selecting ″TCGA PanCancer Atlas Studies″. Expression levels were derived from TCGA data sets processed with the RNA-Seq by Expectation-Maximization (RSEM) pipeline. Furthermore, a correlation study of copy number variations, methylation, mutations with RNA expression levels, and mutational sites within PCNA domains was also determined.

### 2.4 Survival Prognosis

The survival prediction for PCNA across all TCGA tumors was retrieved using GEPIA2 (http://gepia2.cancer-pku.cn/#index) where gene expression was quantified in normalized transcripts per million (nTPM) based on TCGA and GTEx data sets.^41^ The gene of interest was selected, and patients were stratified into two groups, high and low expression, utilizing a 50% (median) cutoff range. Kaplan–Meier (KM) survival curves were generated for each group, and statistical significance was evaluated by implementing the log-rank test. The ″Survival Map″ module of GEPIA2 was employed to collect data on overall survival (OS) and disease-free survival (DFS) in all malignancies, associated with PCNA expression.

### 2.5 Immune Microenvironment Characterization

The Tumor-Immune System Interaction Database (TISIDB) (http://cis.hku.hk/TISIDB/) was used to study the relationship between PCNA expression and the immune microenvironment in pan-cancer. It is a user-friendly web platform that incorporates diverse oncoimmunology data, including as high throughput screening data, molecular profiles of immunotherapy patients, multiomics data from the TCGA, literature, and public databases.^42^ The Spearman correlation between PCNA expression and lymphocytes, immunoinhibitory proteins, and chemokines, was assessed and visualized using heatmaps.

### 2.6 Gene Expression and Drug Sensitivity

Gene Set Cancer Analysis (GSCA) (https://guolab.wchscu.cn/GSCA/) is a user-friendly web-based tool that facilitates integrated exploration of the genomics, immunogenomics, and pharmacogenomics attributes of gene sets across tumor types.^43^ It encompasses over 10,000 multidimensional genomic information from 33 cancer types provided by the TCGA. It has four modules: “Expression”, “Immune”, “Mutation”, and “Drug”.^43^ GSCA also includes data from a total of 1861 cell lines across both data sets and features approximately 750 small-molecule drugs from Genomics of Drug Sensitivity in Cancer (GDSC) and Cancer Therapeutics Response Portal (CTRP).^43^ The drug module assisted to determine the relationship between PCNA mRNA expression (RSEM normalized) and drug sensitivity (IC_50_) across pan-cancer. The *P*-value was corrected using false discovery rate (FDR) < 0.05.

### 2.7 Functional Annotation Analysis

The CancerSEA database (http://biocc.hrbmu.edu.cn/CancerSEA/) was used to examine 14 distinct functional states, including stemness, invasion, metastasis, proliferation, epithelial–mesenchymal transition (EMT), angiogenesis, apoptosis, cell cycle, differentiation, DNA damage, DNA repair, hypoxia, inflammation, and quiescence, in cancer cells at single-cell resolution.^44^ The database contains information from 41,900 cancer cells from 25 cancer types.^44^ Average correlations between PCNA expression (TPM/counts per million (CPM)) and these functional states across multiple tumors were observed, and the results were visualized using a bubble plot.

### 2.8 Protein–protein Interaction (PPI) Network and Gene Enrichment Analysis

Search Tool for the Retrieval of Interacting Genes/Proteins (STRING) version 12.0 (https://string-db.org/) is a database of 59,309,604 proteins spanning 12,535 species with predicted protein–protein interactions.^45^ It retrieves interaction data from databases, mining texts, conserved coexpression data, high-throughput experimental data, and genomic context predictions for query proteins.^45^ PCNA was searched, and the maximum interactor was set to 50 with a high confidence level (0.7). The network was imported to Cytoscape which facilitates the visualization of complex network.^46^ GSCA tool was utilized for the analysis of differential gene set variation (GSCA) using TCGA samples.^43^ The interactors were analyzed for functional enrichment using the ShinyGo 0.81 version (http://bioinformatics.sdstate.edu/go/) using the default settings. It is a Shiny graphical web application built with many R/Bioconductor programs and a large annotation and network database sourced from Ensembl and STRING-db.^47^

## 3 Results

### 3.1 Pan-Cancer Profiling of PCNA Expression

Using TNMplot,^35^ we found that PCNA mRNA levels were significantly higher (*p* < 0.05) in 21 cancer types compared to their respective normal tissues (Figure 1a). PCNA expression (mRNA) was also assessed across tumor cell line types by HPA,^36^ which demonstrated low cancer specificity (Figure 1b). Lymphoma cell lines had the highest mRNA expression levels, followed by leukemia cell lines (Figure 1b). In contrast, low transcript (mRNA) levels were seen in uterine and gall bladder cancer cell lines (Figure 1b).

**Figure 1:**
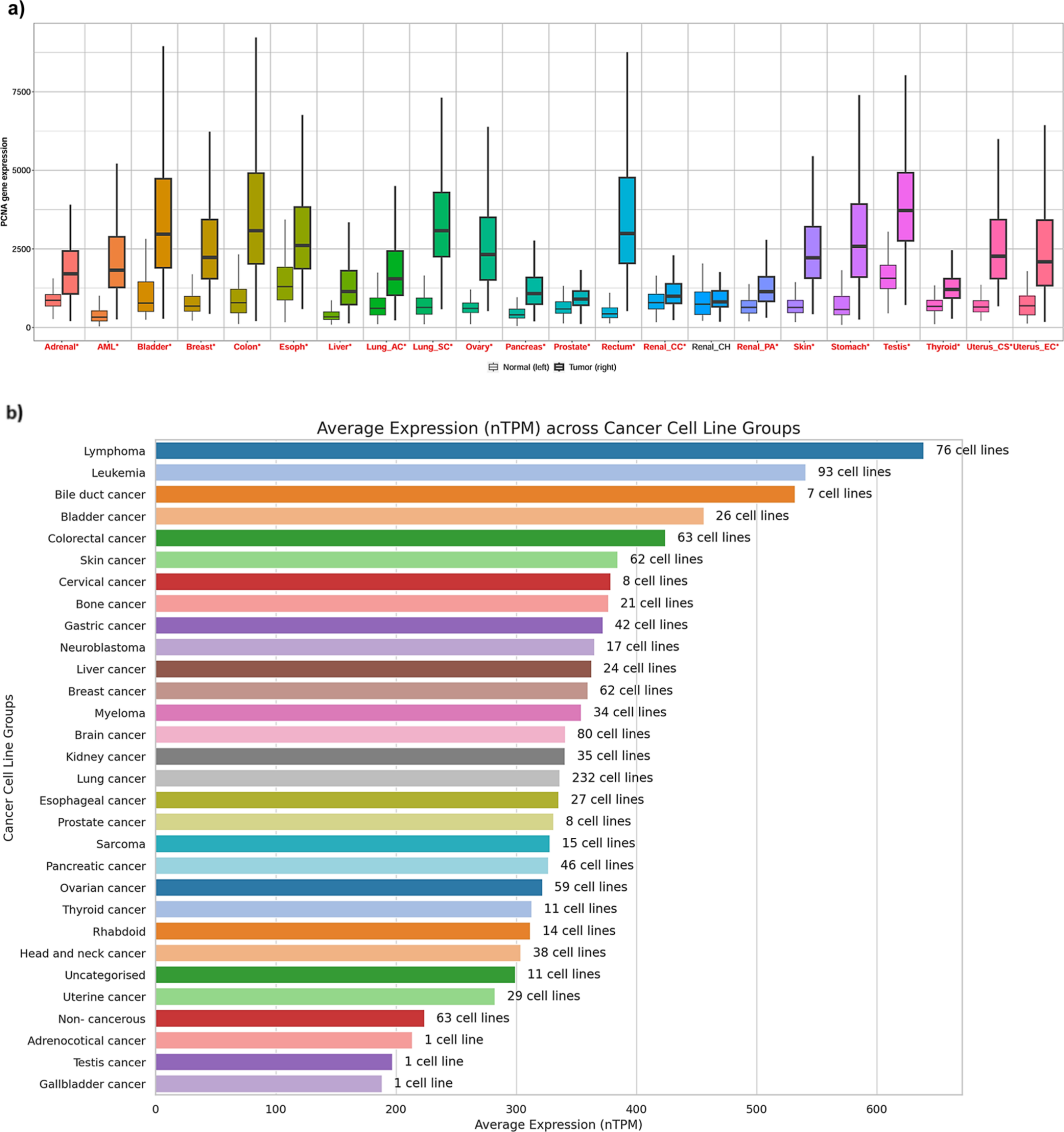
(a) PCNA expression (mRNA) analysis across pan-cancer and corresponding normal tissues (Red color = *p* < 0.05, expression >10 in normal or tumor tissues), including adrenal, acute myeloid leukemia (AML), bladder, colon, esophagus, liver, lung adenocarcinoma (Lung_AC), lung squamous cell carcinoma (Lung_SC), ovary, pancreas, prostate, rectum, renal cell carcinoma, renal papillary adenocarcinoma, skin, stomach, testis, thyroid, uterus carcinosarcoma (Uterus_CS), and uterus endometrial cancer (Uterus_EC). (b) Bar plot of PCNA transcripts level (mRNA) measured by RNA-seq in different cancer cell line categories.

### 3.2 Mapping Pan-Cancer Expression of PCNA in Cancer Pathways

To explore the broader functional landscape of PCNA beyond its classical role in DNA replication and repair, we examined the association between elevated PCNA levels and deregulation of key cancer-associated pathways. These included canonical pathways like p53/Rb and mTOR, where PCNA involvement is anticipated, as well as less directly related pathways such as WNT, NRF2, MYC/MYCN, SWI/SNF, and **HIPPO**, which may reflect coregulated tumorigenic programs or stress-adaptive mechanisms, using the UALCAN portal (Figure 2).^37,38^ The integrated bar plot was constructed using the median Z-score values as the quantitative measure of alteration magnitude in R software.^39^ Substantial variation among cancer types, with altered pathway status (red) correlated with higher expression of PCNA than unaltered ones (others). For instance, in breast and lung cancer, high PCNA levels were associated with WNT pathway alterations, whereas altered mTOR signaling was associated with high pCNA levels in breast, ovarian, and lung cancers. Elevated PCNA was also associated with p53/Rb pathway aberrations in breast cancer and uterine corpus endometrial carcinoma (UCEC). In pancreatic cancer, decreased levels of PCNA were linked with alterations in the NRF2 pathway, while alterations in the MYC/MYCN and HIPPO pathways was linked with increased PCNA levels in lung and pancreatic cancer. Likewise, glioblastoma and head-neck cancers showed similar trends in chromatin modifiers, while breast and ovarian cancers had heightened PCNA levels in the SWI/SNF pathway.

**Figure 2:**
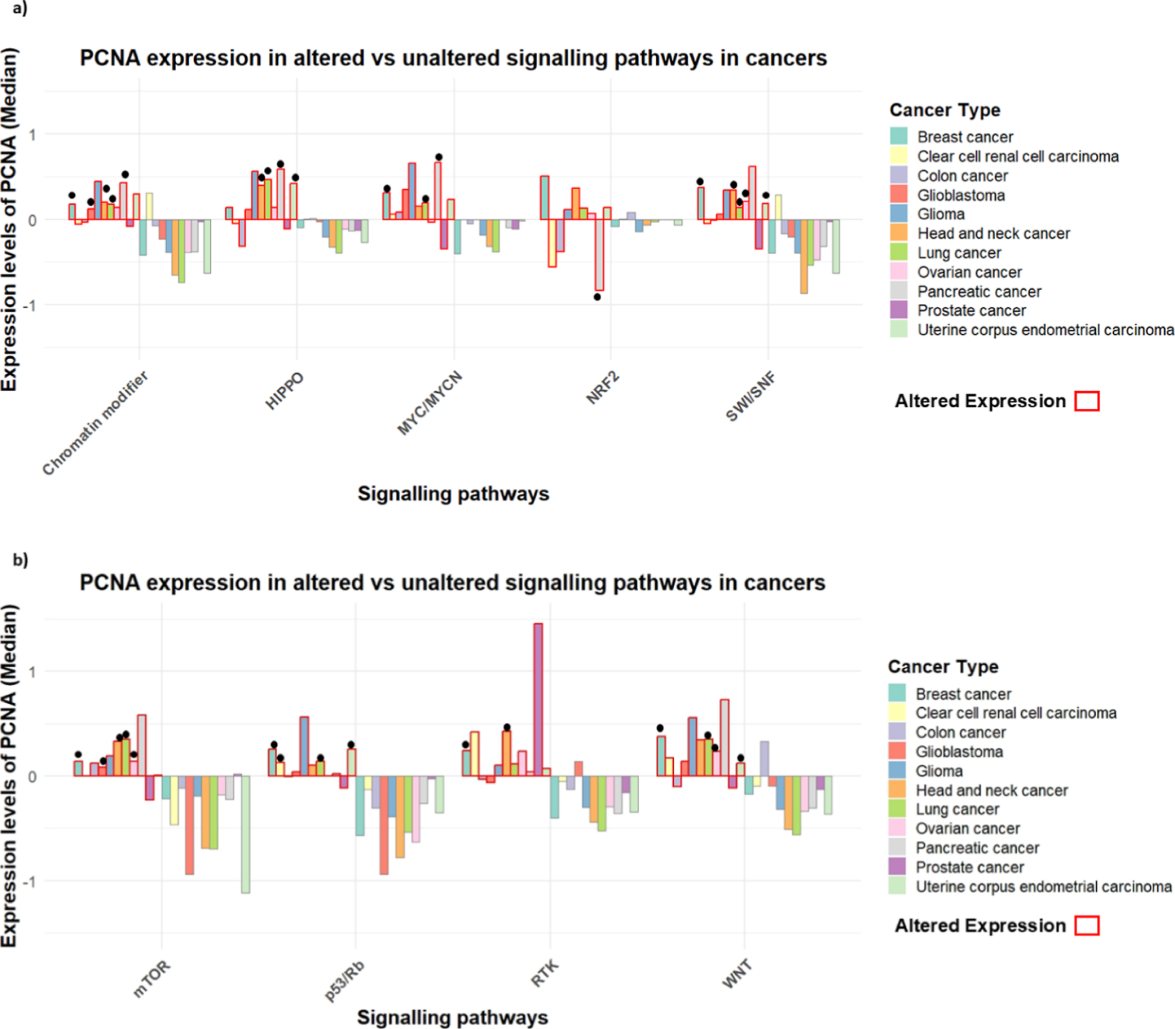
(a,b) Integrated bar plots of PCNA expression levels (median based z scores) across pan-cancer in altered (red highlighted) vs non altered signaling pathways based on somatic alterations status involving integrated proteomics, CNA, and whole-exome data. Black dot represents the statistical significance between the altered and unaltered (others) pathways, *p* < 0.05. Red highlighted bars are the altered signaling pathways in comparison to nonaltered signaling pathways­(others). Cancer types are represented in different color types as showcased in legend.

### 3.3 Genetic Alteration Analysis of PCNA

The genetic variation in PCNA in diverse tumor subtypes from TCGA cohorts was investigated using cBioPortal. The frequency of PCNA genetic alterations was highest in ovarian epithelial tumors (>4%). In this particular tumor subtype, amplification contributed more than half of the variability. Furthermore, mutation was shown to be the predominant form of genetic modification in endometrial cancer, bladder cancer, melanoma, and colorectal cancer whereas amplification was the primary genetic alteration in ovary epithelial tumors, esophagogastric cancer, nonsmall-cell lung cancer (NSCLC), and sarcoma (Figure 3a). Mutation sites were observed throughout the PCNA domains, ranging from amino acids 0 to 261. The mutation sites included 33 missense, 5 truncating, and 2 splice mutations. Variants including D86N/Y, S76F/P, A67P/T, and M244I/R were also frequently observed (Figure 3b). The analysis further revealed that PCNA mRNA expression is significantly influenced by Copy number alterations (CNAs), methylation, and mutations. CNAs show that deletions lower expression, while gains and amplifications increase it (Figure 3c). Mutation analysis revealed that missense mutations slightly increased mRNA expression, while no mutations associated with higher levels (Figure 3d). Methylation showed an inverse correlation with expression, where hypomethylated samples exhibited elevated mRNA levels, highlighting epigenetic regulation (Figure 3e). These findings suggest that genetic alterations in PCNA are relatively rare and may not be the primary driver of its high expression in cancers.

**Figure 3:**
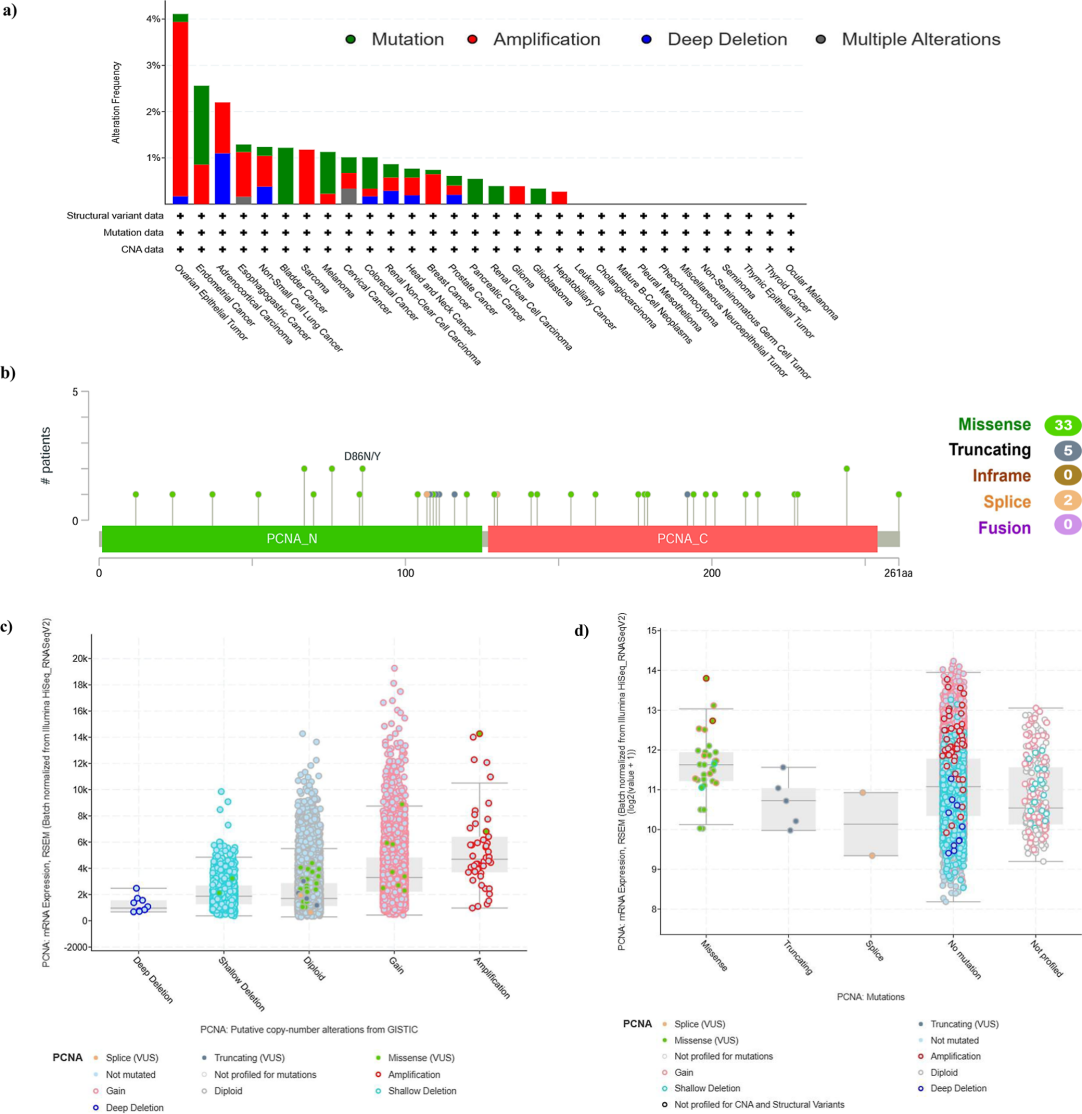

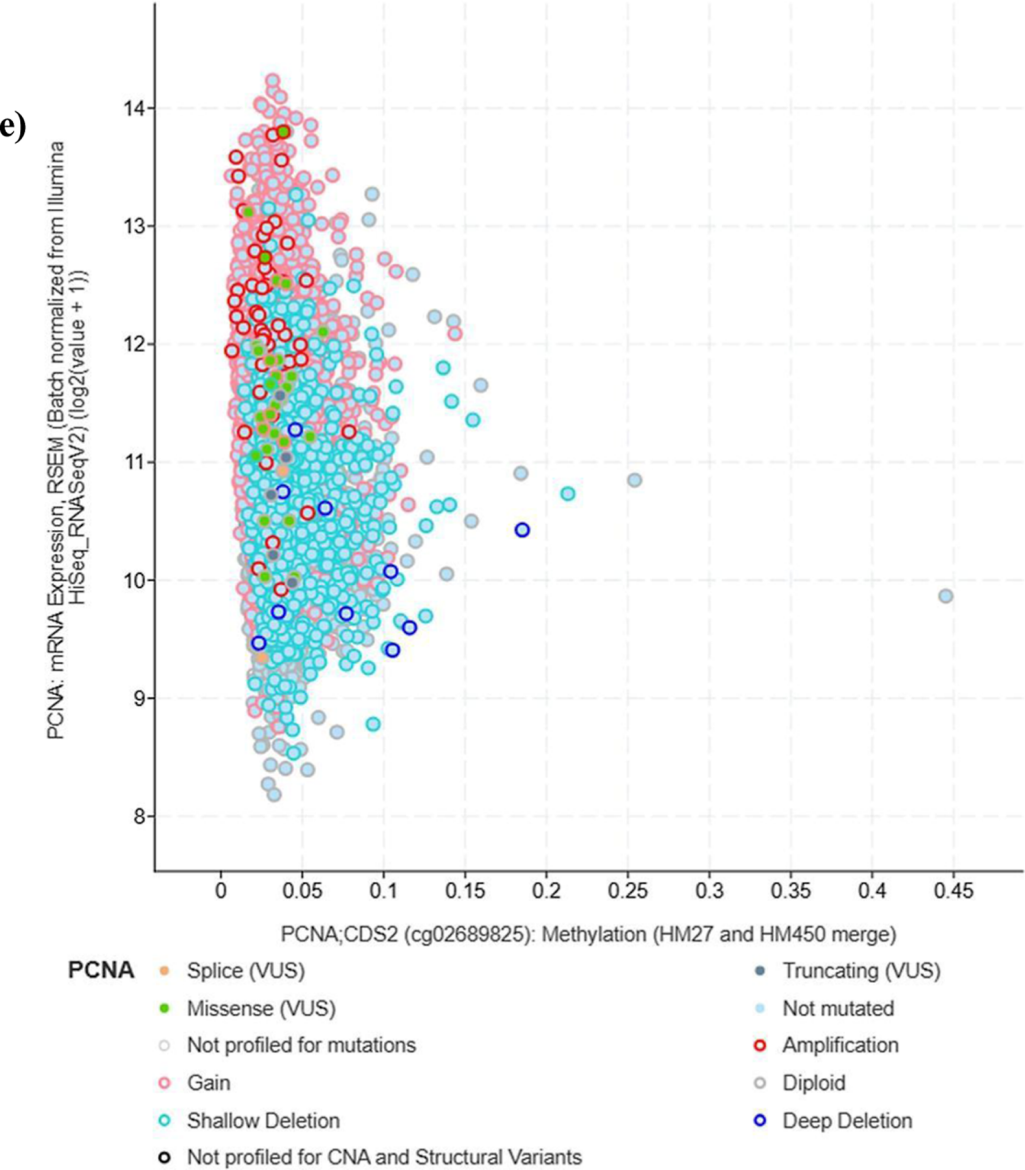
Mutation characteristics of PCNA gene across multiple tumors in the TCGA cohorts using cBioPortal via RNA-Seq V2 data (Illumina HiSeq platform), and are batch-normalized. (a) Frequency of PCNA genetic alterations in TCGA patient groups in pan-cancer. (b) Mutation sites from 0 to 261 amino acids in PCNA domain. (c) Box plot illustrating PCNA mRNA expression and copy number alterations. (d) PCNA-mRNA expression and various mutations. (e) PCNA-mRNA levels and methylation status.

### 3.4 Survival Prognosis of PCNA Expression in Pan-Cancer

The impact of PCNA gene expression in patient survival outcomes in various tumor types was examined. Our investigation demonstrated that high PCNA gene expression exhibited a correlation with poor OS in Adrenocortical carcinoma (ACC) (HR = **3.7**, *p* = **0.002**), kidney renal papillary cell carcinoma (KIRP) (HR = **2.2**, *p* = **0.014**), brain lower grade glioma (LGG) (HR = **2.6**, p = **1.4 × 10** ^**–6**^), lung adenocarcinoma (LUAD) (HR = **1.6**, *p* = **0.0042**), mesothelioma (MESO) (HR = **2.4**, *p* = **0.00047**), sarcoma (SARC) (HR = **1.7**, *p* = **0.0092**), and uveal melanoma (UVM) (HR = **3**, *p* = **0.031**) (Figure 4a). Inversely, high expression of PCNA was linked to improved prognosis in patients with cervical squamous cell carcinoma and endocervical adenocarcinoma (CESC) (HR = 0.58, *p* = **0.023**) and thymoma (THYM) (HR = 0.09, *p* = **0.026**) (Figure 4a). Additionally, high PCNA expression and shorter DFS in adrenocortical carcinoma (ACC) (HR = **2.1**, *p* = **0.032**), brain lower grade glioma (LGG) (HR = **1.8**, *p* = **0.00033**), liver hepatocellular carcinoma (LIHC) (HR = **1.4**, *p* = **0.017**), and mesothelioma (MESO) (HR = **1.9**, *p* = **0.021**) patients was observed (Figure 4b). Conversely, high expression of PCNA was associated with favorable outcome in stomach adenocarcinoma (STAD) (HR = 0.52, *p* = **0.0013**) (Figure 4b).

**Figure 4:**
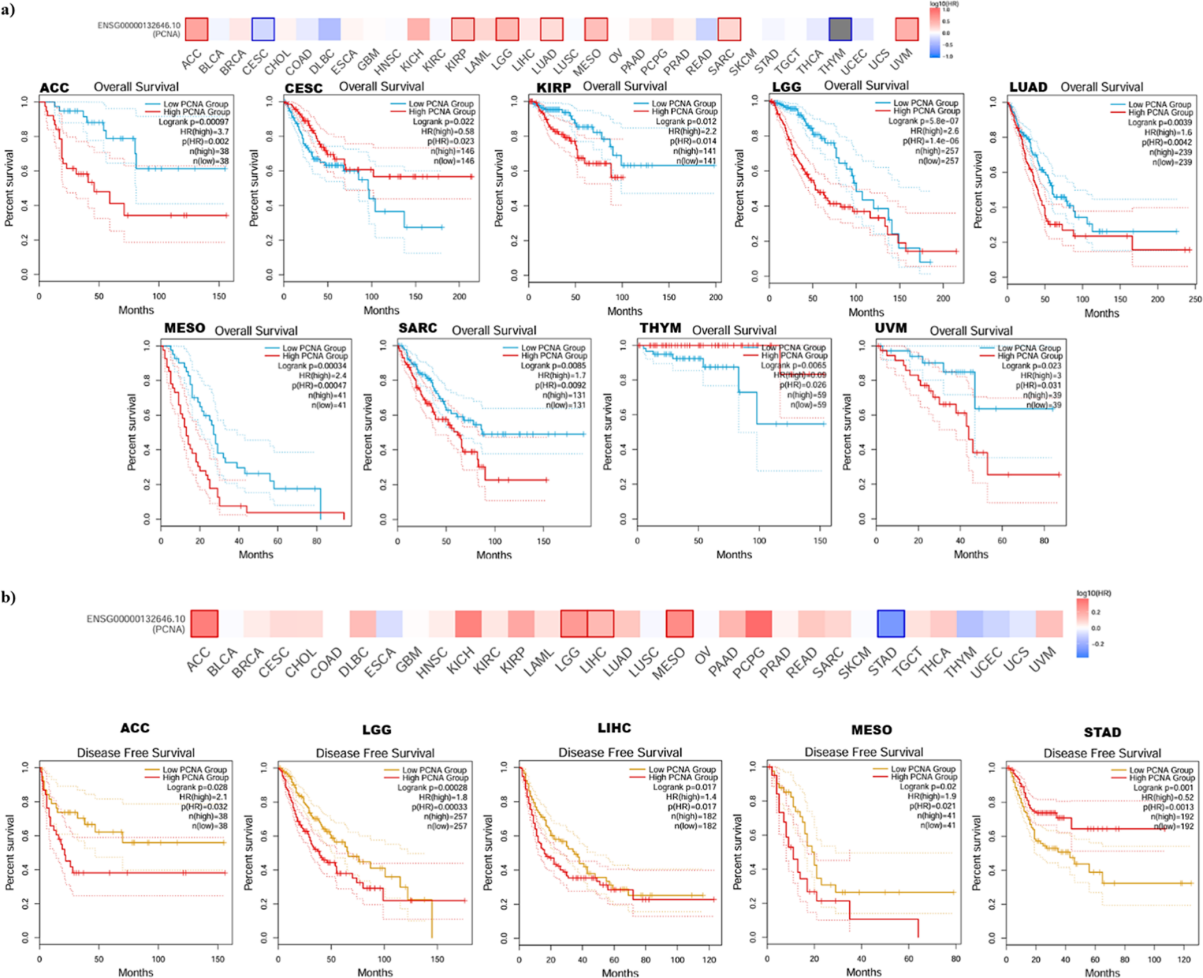
Survival analysis based on PCNA mRNA expression was performed in GEPIA2 using nTPM values from TCGA/GTEx data. Patients were divided into high and low expression groups using the 50% (median) cutoff. (a) Survival maps and Kaplan–Meier overall survival outcomes for PCNA mRNA expression. (b) Survival maps and Kaplan–Meier disease free survival outcomes for PCNA mRNA expression.

### 3.5 PCNA Expression and Immune Microenvironment Characterization

TISIDB^42^ was used to investigate the association between PCNA gene expression and the immune microenvironment in pan-cancer, encompassing tumor-infiltrating lymphocytes (TILs), immuno-inhibitors, and chemokines. Figure 5a demonstrates that activated helper T cells (CD4+T cells) and active cytotoxic T lymphocytes (CD8+T cells) strongly correlate with PCNA expression across multiple malignancies. In contrast, regulatory T cells (Treg), macrophages, and eosinophils revealed a negative correlation with cancer types such as breast invasive carcinoma (BRCA), colon adenocarcinoma (COAD), glioblastoma multiforme (GBM), head and neck squamous cell carcinoma (HNSC), kidney chromophobe (KICH), and ovarian serous cystadenocarcinoma (OV). In Figure 5b, several immune checkpoint markers, such as PDCD1, and CTLA-4, display significant positive correlations with PCNA levels in multiple cancer types, including uveal melanoma (UVM), thyroid carcinoma (THCA), kidney renal clear cell carcinoma (KIRC), and head and neck squamous cell carcinoma (HNSC). Remarkably, cancers such as brain lower grade glioma (LGG), mesothelioma (MESO), uveal melanoma (UVM), and liver hepatocellular carcinoma (LIHC), which exhibit elevated PDCD1 levels, are also associated with poorer OS and DFS. This suggests that PDCD1 upregulation may contribute to tumor progression and immune evasion in these malignancies, although further functional studies are required to establish causality. Conversely, some markers, such as KDR (endothelial cells), CD160 (NK cells and cytotoxic T cells), CSF1R (macrophages and mononuclear phagocyte systems), and TGFB1 (T cells, dendritic cells, stromal, and immune cells), indicated a negative association with PCNA expression in breast invasive carcinoma (BRCA), lung adenocarcinoma (LUAD), stomach adenocarcinoma (STAD), along with various malignancies. In Figure 5c, a notable positive correlation among chemokines like CXCL10 CXCL11 with PCNA expression levels across pan-cancer was observed; however, other chemokines such as CCL14 and CXCL12 displayed a negative correlation with most cancers. This suggests a complex and dynamic immune microenvironment, where pro-tumorigenic and tumor-suppressive signals interact, potentially influencing immune evasion, inflammation, and tumor progression in a tissue-specific manner, with markers and chemokines behaving as either tumor-progressive factors or mediators of antitumor immunity.

**Figure 5:**
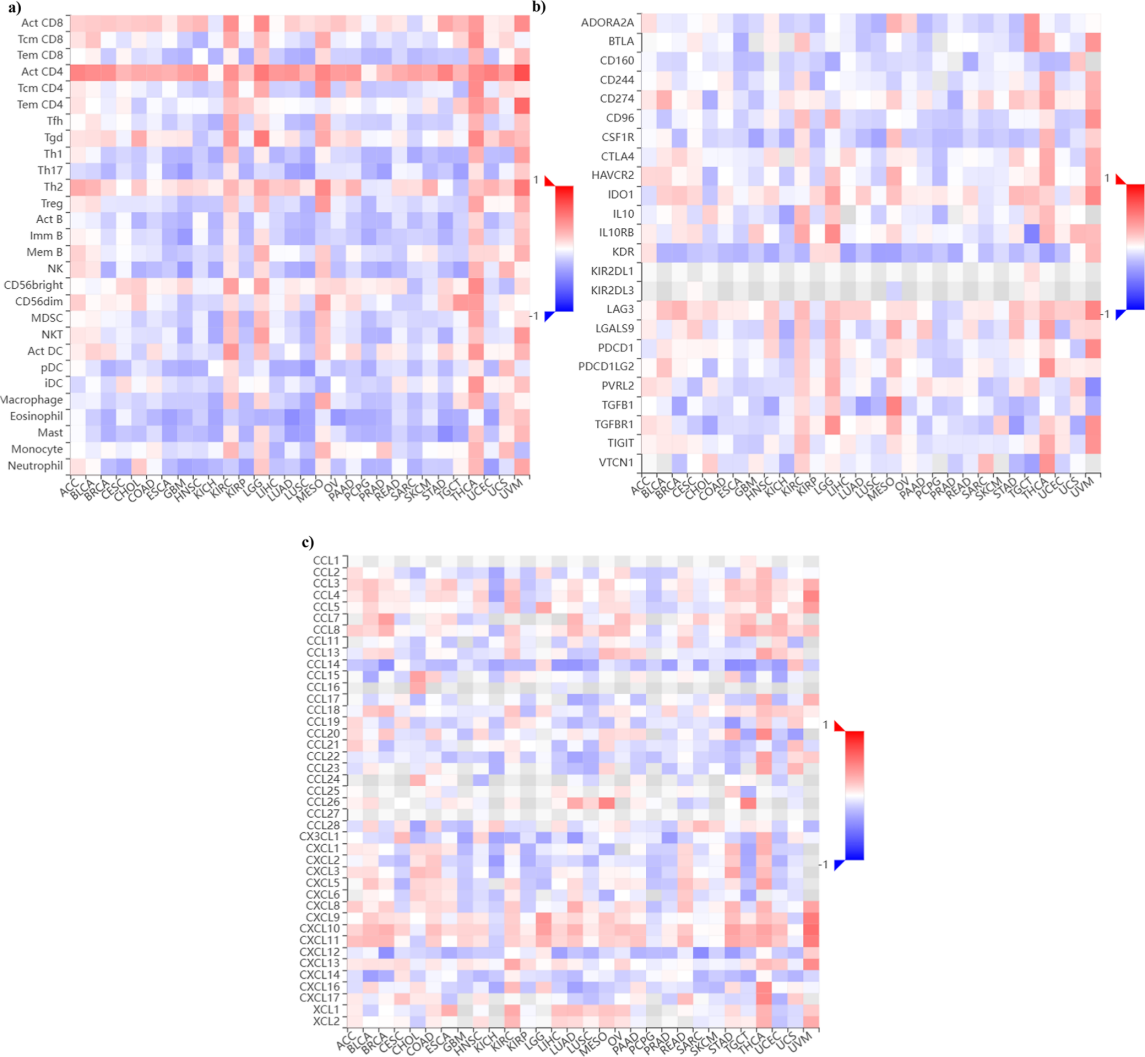
PCNA mRNA expression and immune cell infiltration analysis using TISIDB web server (Red: positively correlated, Blue: negatively correlated). Heatmap associating (a) tumor infiltrating lymphocytes (b) immunoinhibitors (c) chemokines with PCNA mRNA expression.

### 3.6 PCNA Expression and Drug Sensitivity

The GSCA web server was utilized to investigate the correlation between PCNA mRNA expression and drug sensitivity (IC_50_) of the top 30 therapeutic compounds in pan-cancer. In Figure 6a (GDSC drug sensitivity), the majority of drugs, such as Navitoclax, NPK76-II-72-1, and GSK1070916, showed a significant negative association (FDR ≤ 0.05) with PCNA expression, indicating that elevated expression of the gene may increase drug sensitivity. Nevertheless, drugs such as Trametinib, RDEA119, and Selumetinib demonstrated a positive relationship with PCNA expression, signifying therapeutic resistance in cancer. The bubble plot produced from the CTRP data set revealed PCNA as a key gene of interest. The top 30 drug molecules, including Ciclopirox, CD-437, and Gemcitabine, displayed a substantial negative correlation with FDR ≤ 0.05, establishing drug sensitivity with higher gene expression levels (Figure 6b).

**Figure 6:**
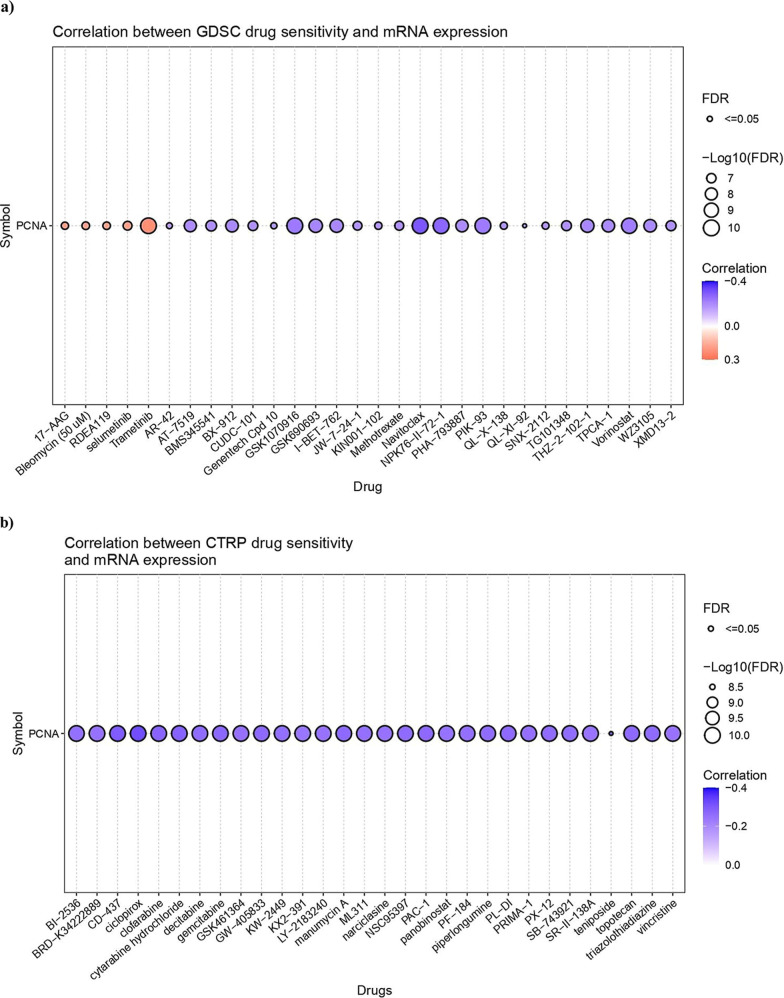
Bubble plot illustrating PCNA mRNA expression (RSEM normalized) for (a) GDSC drug sensitivity IC_50_ (top 30 molecules) (b) CTRP drug sensitivity IC_50_ (top 30 molecules) across pan-cancer.

### 3.7 Functional Annotation Analysis of PCNA in Pan-Cancer

The CancerSea analysis of PCNA underscores its critical involvement with biological processes across multiple tumor types at single-cell resolution. The bubble plot reveals that processes like cell cycle, DNA repair, and invasion positively correlate with PCNA expression, implying its central role in tumor growth and genomic instability, while its correlation with inflammation and EMT varies by tumor, indicating its influence on tumor microenvironment and metastasis (Figure 7). Furthermore, it demonstrated a negative correlation with functional states such as quiescence, inflammation, and hypoxia, reflecting a varied link depending on cancer type (Figure 7). For instance, in most malignancies, including breast invasive carcinoma (BRCA), lung adenocarcinoma (LUAD), and chronic myelogenous leukemia (CML), PCNA expression is strongly linked to cell cycle, DNA damage, EMT, and proliferation. In contrast, malignancies such as high grade glioma (HGG), nonsmall-cell- lung cancer (NSCLC), and colorectal cancer (CRC) showcased a negative relationship of PCNA levels with angiogenesis, apoptosis, hypoxia, and metastasis. These findings underline PCNA’s multifaceted role in cancer development, which may differ depending on the cancer-type context.

**Figure 7:**
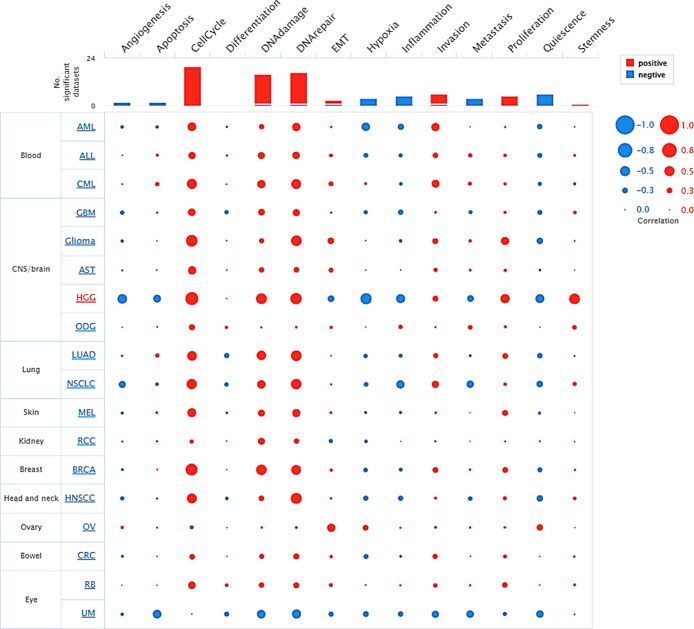
Bubble plot showcasing functional annotation relationship with PCNA mRNA expression (TPM/CPM) at single cell level in pan-cancer.

### 3.8 PPI Network and Gene Enrichment Analysis of PCNA

Understanding PCNA’s interactome is essential due to its critical role in DNA replication, repair, cell cycle regulation, invasiveness, and stemness offering deeper insights into tumor biology. The PPI network of PCNA, and its top 50 binding proteins, was generated using STRING data (high confidence, 0.7) and visualized in Cytoscape to ascertain its engagement in complex regulatory mechanisms involving interactors that contribute to tumor progression (Figure S2a). Key PCNA binding partners include CDK1A, FEN1, MSH6, LIG1, RFC, and various DNA polymerases, which provide insight into the regulatory processes, pathway involvement, therapeutic targets, and disease associations. Additionally, these PCNA and its interactors were aimed to evaluate the integrated expression across TCGA cancer types using GSVA scores. The boxplot displays significantly higher expression levels of the PCNA-associated signature in tumor samples (red) compared to normal tissues (blue) across multiple cancers such as breast invasive carcinoma (BRCA), head and neck squamous cell carcinoma (HNSC), lung squamous cell carcinoma (LUSC), and thyroid carcinoma (THCA) (Figure S2b). The PCNA interactors were subsequently evaluated for gene ontology (GO) enrichment, with the findings shown as interactive plots. Figure S2c reveals biological processes pertaining to our gene set, including “DNA strand elongation involved in DNA replication”, “Translesion synthesis”, and “Post replication repair” emphasizing their importance in maintaining genomic stability and responding to DNA damage. Figure S2d highlights the enriched components for cellular localization of the relevant proteins, including “DNA replication factor C complex”, “Ctf 18RFC-like complex”, and “DNA polymerase complex”. In Figure S2e, enriched molecular functions such as “DNA clamp loader”, “DNA-directed DNA polymerase activity”, and “Single-stranded DNA helicase activity” underscore the significance of biochemical roles in preserving cellular integrity. Furthermore, the heatmap of curated pathway enrichment results derived from KEGG, Reactome, and Wiki Pathways modules of STRING tool elucidated critical mechanisms involved like “ATM signalling pathway”, “JAK-STAT signalling pathway” “MAPK6/MAPK4 signaling”, “PI3K-Akt signaling pathway”, “Wnt signaling”, “p53 signalling pathway” were strongly related to PCNA partners (Figure S 2f). Collectively, these analyses offer a comprehensive functional landscape of PCNA’s interaction network, highlighting its involvement in key oncogenic pathways and potential as a therapeutic target in cancer progression.

## 4 Discussion

In this study, we used a multiomics strategy to investigate the potential of PCNA as a prognostic biomarker using pan-cancer analysis. PCNA mRNA expression was elevated in 21 of the 22 cancer types represented in the TNM plot (Figure 1a), consisting of bladder, breast, colon, lung, ovary, rectum, stomach, and testis. The majority of the outcomes are well aligned with recent published reports^22–24,30,34,48,49^ whereas renal chromophobe (Renal_CH), a rare subtype of renal carcinoma, has lower PCNA expression, which requires additional validation. Also, PCNA transcript levels (mRNA) of cancer cell lines in the HPA database were examined by incorporating a common approach utilized in other investigations^50,51^ to bring about a relationship of the gene as a pan-cancer target. PCNA mRNA expression manifested low cancer specificity, ranging from high concentrations in lymphoma to moderate levels in gastric cancer and lower amounts in adrenocortical cancers, indicating that it is abundantly expressed in multiple cancers rather than a single malignancy (Figure 1b), thereby rendering it a strong candidate for prognostic biomarker. Our findings from pan-cancer analysis support the hypothesis that PCNA’s expression (mRNA) across diverse cancers could be harnessed for broad-spectrum cancer diagnostics. Furthermore, variable expression can be attributed to genetic mutations, post-translational modifications, epigenetic changes, altered signaling networks, and the tumor microenvironment, which may contribute to cancer development and survival.

Canonical and noncanonical signaling pathways such as Notch, Wnt, mTOR, and p53 are predominantly altered in cancers. Given that the PCNA orchestrates many cellular regulating processes, it is implausible to overlook that its expression will be impeded in these signaling systems. With this rationale, an attempt was made to evaluate the PCNA levels in altered and nonaltered pathway statuses (WNT, mTOR, p53/Rb, RTK, NRF2) using the UALCAN database which incorporated protein expression (using mass spectrometry–based proteomics) and correlate these protein data with DNA mutations, copy number alterations, and mRNA expression levels. PCNA levels were substantially elevated in signaling pathways in most tumor types, signifying oncogenic synergy between mechanistic dysregulation and proliferative ability mediated by PCNA. Wang et al. showcased that PCNA serves as an oncogene in lung cancer by upregulating STAT3, enhancing cell proliferation, formation of clonal cells, carcinogenesis, and apoptosis inhibition.^52^ Similarly, phosphorylated PCNA at Tyr-211 to Asp-211 substitution increased cancer development through EMT and aggressive cell migration by upregulating ATM/Akt/GSK3β/Snail signaling cascade.^53^ The differential transcriptome levels of PCNA across tissues, cell lines, and regulatory cascades prompted us to speculate what causes such overexpression in malignancies. As a result, somatic mutation patterns using cBioPortal were examined. The genetic aberration in PCNA were more prevalent in ovarian epithelial tumors, followed by endometrial cancer and adrenocortical carcinoma (Figure 3a). Amplification was the most common genetic event across several types of cancer, while other changes like mutation and deep deletion were also detected. Consequently, our data suggest a tumor-type-specific preference for genetic variations in PCNA, which might correspond to distinctive carcinogenic pathways in various malignancies. Additionally, hypomethylation and PCNA mRNA levels have a reciprocal relationship (Figure 3e), underscoring the importance of epigenetic control in modifying PCNA activity and tumor growth. More broadly, PCNA functions as a key platform for recruiting and coordinating epigenetic modifiers, including DNA methyltransferases such as DNMT1. Misregulation of these interactions, particularly in the absence of regulatory proteins like p21 can lead to aberrant methylation patterns and genomic instability, highlighting PCNA’s central role in the interface between replication and epigenetic maintenance.^54^ Supporting the epigenetic implications observed in our study, previous research has demonstrated that disruption of the DNMT1/PCNA/UHRF1 complex leads to widespread DNA hypomethylation, which acts as a driving factor in the development of various human cancers. This loss of methylation marks triggers specific epigenetic and genetic changes commonly seen in tumors and initiates key steps in cancer formation. The integrity of the DNMT1/PCNA/UHRF1 complex is vital for faithfully maintaining DNA methylation patterns during cell division, and its disturbance significantly contributes to malignant transformation. Given the limited frequency of PCNA genetic alterations, its overexpression in cancers is likely driven by deregulated signaling pathways rather than direct genomic alterations.^55^

According to our findings, PCNA expression illustrated atypical prognostic values in pan-cancer; for example, in adrenocortical carcinoma (ACC), high PCNA levels were linked to poor OS (Figure 4a), whereas, in thymoma (THYM), elevated PCNA expression contributed to better OS (Figure 4a). It is also pertinent to point out that in the prognostic analysis of the same tumor (adrenocortical carcinoma, brain lower grade glioma, mesothelioma), the OS and DFS were comparable (Figure 4b). Previous experimental studies have established PCNA as a prognostic factor across various cancer types,^52,56–58^ supporting our pan-cancer analysis that highlights its context-dependent prognostic value. As the survival data suggests PCNA as a prognosticator, it emerges as an appealing therapeutic target in cancer treatment. Current developments provide persuasive evidence to establish PCNA as the druggable target, with studies demonstrating its selective targeting in cancer cells.^59,60^ For instance, small-molecules inhibitors of PCNA such as ATX-101 and AOH1996 are currently undertaking phase I clinical testing for anticancer therapy without any harmful effects on normal healthy cells.^60–62^

The immune microenvironment is an integral component of the tumor microenvironment. Hence, immune infiltration analysis becomes crucial in pan-cancer studies because it offers insights into how specific gene interacts with immune cell types such as T-cells, B-cells, macrophages, dendritic cells, and fibroblasts to foster or repress cancer progression. Growing evidence hints that PCNA has a function in modulating immunological responses.^63–66^ Immune microenvironment characterization revealed a paradoxical immune landscape in which immune intrusion exists but is downregulated, implying that tumors might resort to alternative immune evasion strategies, such as immune checkpoint activation (PDCD1, CTLA4), for example, in head and neck squamous cell carcinoma (HNSC), kidney renal clear cell carcinoma (KIRC), mesothelioma (MESO), thyroid carcinoma (THCA), and uveal melanoma (UVM) (Figure 5a–c). Our findings emphasize the dearth of functional validation and the need for further investigation into how to target immune checkpoints in PCNA-driven malignancies or combinatorial drugs as immunotherapy to boost clinical responses.

Drug resistance is challenging to mitigate due to the disparity of individuals and malignant tumors, as well as cancer’s ability to bypass treatments. Considering this perspective, the data inspected using GDSC and CTRP data set in GSCA across pan-cancer showed quite promising results demonstrating increased mRNA levels of PCNA are related to increased drug sensitivity to therapeutic agents like Navitoclax, Ciclopirox, PIK-93 (Figure 6a,b) which primarily have pro-apoptotic, antifibrotic, PD-L1 modulation, and antiproliferation properties.^67–69^ Nonetheless, very few drug compounds displayed resistance to high PCNA expression, reflecting that PCNA-driven tumors might be exploiting alternative mechanisms to sustain. Overall, the outcome reinforces PCNA as a biomarker for predicting drug response. Yet our study emphasized the paucity of research data to substantiate our findings, which could otherwise provide new avenues for researchers to understand the drug response. To delve deeper into functional aspects, sc-RNA seq analysis of the PCNA gene, particularly in tumor biology was performed. Despite its fundamental role in DNA metabolism and repair processes, the results highlighted that the gene is strongly correlated with DNA damage, invasion, and proliferation and is moderately associated with EMT and stemness in various malignancies (Figure 7), indicating context-dependent relationships; either positive or negative correlation for the same functional aspect in cancers, and a broader influence on tumor microenvironment. It specifies that PCNA executes a multifaceted function in cancer development and progression, although further investigations are needed to grasp the tumor-specific mechanisms completely. In addition to this, enrichment analysis was employed to construct and visualize PPI network, and top 50 genes with most significance was identified, suggesting PCNA along with its binding partners are involved in critical pathways to maintain genome stability. Furthermore, the heightened GSVA scores of PCNA and its interactors indicates that the PCNA related gene signatures are upregulated in several malignancies compared to normal ones (Figure S2b). Since PCNA and its partners plays prominent roles in DNA replication, repair, and cell cycle progression evident in the enrichment data (Figure S2c–e), the integrated upregulation of these genes implies amplified proliferative activity and genomic instability in cancer development. This interpretation is consistent with the role of dysregulated cell cycle regulators, such as CDC20-M, in driving chromosomal instability in cancer.^70^ Additionally, the curated pathway enrichment heatmap emphasized the involvement of PCNA clients in key oncogenic signaling cascades across multiple databases, reinforcing their role in tumorigenesis.

Although multiple multiomics data on PCNA across pan-cancers has been examined in our study, certain limitations can also be oriented including small sample size in a few malignancies may have resulted in inaccurate results. Moreover, the study is based on preliminary findings associating PCNA to cancer development. Furthermore, our work highlights the deficiencies of experimental investigations on epigenetic regulation, particularly the significance of hypomethylation in PCNA-driven malignancies. It also emphasizes the dynamics between PCNA transcriptome levels and immune cell intrusions. Besides, there is limited clinical evidence to support drug response and PCNA expression in order to develop combinatorial therapies where traditional treatments are insufficient.

## 5 Conclusion

In conclusion, PCNA emerges as a co-oncogene and an indispensable pan-cancer prognostic biomarker in our investigation. It is consistently upregulated in diverse tumors and cell line categories. Heightened PCNA concentration correlates substantially with patient prognosis, immune cell infiltration, cancer-associated altered signaling cascades, functional states, and therapeutic sensitivity. The present study is a pioneering effort to conduct a pan-cancer analysis on PCNA, aiming to holistically comprehend the multidimensional role of PCNA in cancer pathology and advocate it as a potent therapeutic candidate for synergistic therapies to enhance intervention and longevity.

## Supplementary Material



## Data Availability

All data sets analyzed in this study were obtained from publicly accessible repositories. The expression profiling data were sourced from TNMplot (https://tnmplot.com/analysis/), Human Protein Atlas (https://www.proteinatlas.org/), and UALCAN (https://ualcan.path.uab.edu/index.html). Genetic alteration data were retrieved from cBioPortal (http://www.cbioportal.org), while survival analysis was conducted using GEPIA2 (http://gepia2.cancer-pku.cn/#index). Immune microenvironment characterization was performed using TISIDB (http://cis.hku.hk/TISIDB/), and drug sensitivity analysis was carried out via the GSCA tool (https://guolab.wchscu.cn/GSCA/). Functional annotation analysis was performed using CancerSEA (http://biocc.hrbmu.edu.cn/CancerSEA/), while protein–protein interaction (PPI) networks were constructed using STRING version 12.0 (https://string-db.org/) and visualized using Cytoscape. Functional enrichment of interactors was conducted using ShinyGo 0.81 (http://bioinformatics.sdstate.edu/go/). The R scripts used for graphical representation are available upon request. Additionally, the Supporting Information files include relevant experimental data generated or processed during this study.

## Notes

Supporting Information for the mRNA expression analysis of PCNA in different cell line categories. (Table S1) Supporting Information for the analysis of mapping pan-cancer expression of PCNA in cancer pathways. (Figure S1 and Table S2) Supporting Information for heatmap interaction of selected pathways and enriched mapped genes from STRING database (KEGG, Wiki pathways, Reactome) for gene enrichment analysis of PCNA. (Table S3) Supporting Information for PPI Network and Gene Enrichment Analysis of PCNA. (Figure S2 a–f) (PDF)

## Abbreviations

ACC: Adrenocortical carcinoma
ALL: Acute lymphoblastic leukemia
AML: Acute myeloid leukemia
AST: Astrocytoma
BLCA: Bladder urothelial carcinoma
BRCA: Breast invasive carcinoma
CESC: Cervical squamous cell carcinoma and endocervical adenocarcinoma
CHOL: Cholangiocarcinoma
CML: Chronic myelogenous leukemia
COAD: Colon adenocarcinoma
CRC: Colorectal cancer
DLBC: Lymphoid neoplasm diffuse large B-cell lymphoma
ESCA: Esophageal carcinoma
GBM: Glioblastoma multiforme
HGG: High grade Glioma
HNSC: Head and neck squamous cell carcinoma
HNSCC: Head and neck cancer
KICH: Kidney chromophobe
KIRC: Kidney renal clear cell carcinoma
KIRP: Kidney renal papillary cell carcinoma
LAML: Acute myeloid leukemia
LGG: Brain lower grade glioma
LIHC: Liver hepatocellular carcinoma
LUAD: Lung adenocarcinoma
LUSC: Lung squamous cell carcinoma
MEL: Melanoma
MESO: Mesothelioma
NSCLC: Nonsmall-cell- lung cancer
ODG: Oligodendroglioma
OV: Ovarian serous cystadenocarcinoma
PAAD: Pancreatic adenocarcinoma
PCPG: Pheochromocytoma and paraganglioma
PRAD: Prostate adenocarcinoma
RB: Retinoblastoma
READ: Rectum adenocarcinoma
RCC: Renal cell carcinoma
SARC: Sarcoma
SKCM: Skin cutaneous melanoma
STAD: Stomach adenocarcinoma
TGCT: Testicular germ cell tumors
THYM: Thymoma
THCA: Thyroid carcinoma
UCEC: Uterine corpus endometrial carcinoma
UCS: Uterine carcinosarcoma
UVM/UM: Uveal melanoma.
