# Supplementary material for: PCNA in Pan-Cancer: A Prognostic Biomarker Unveiled Through a Data-Driven, Multidimensional Analysis of Transcriptomics, Immunity, and Functional Profiling
Source: ACS Omega. 2025 Jun 26;10(26):28311–26. doi: 10.1021/acsomega.5c03331 (PMC12242617; doi:10.1021/acsomega.5c03331)
Supplement: Supplementary file 1 [file ao5c03331_si_001.pdf]

Supplementary Information

PCNA in Pan-Cancer: A Prognostic Biomarker Unveiled Through a Data-Driven, Multidimensional Analysis of Transcriptomics, Immunity, and Functional Profiling

Depanshi Pandit[1], Ravindranath Bilachi Sanganabasappa[2*] Amardeep Dhillon[3], Sanjiban Chakrabarty[4]

1. Department of Biotechnology, Manipal Institute of Technology, Manipal, Manipal Academy of Higher Education, Manipal, Karnataka, India-576104. Email: depanshi.mitmpl2023@learner.manipal.edu

2. Department of Biotechnology, Manipal Institute of Technology, Manipal, Manipal Academy of Higher Education, Manipal, Karnataka, India-576104.
Email: ravindranath.bs@manipal.edu

3. The Institute for Mental and Physical Health and Clinical Translation, School of Medicine, Deakin University, Geelong, Waurn Ponds, Victoria 3216, Australia. Email: amardeep.dhillon@deakin.edu.au

4. Department of Public Health Genomics, Manipal School of Life Sciences, Manipal Academy of Higher Education, Manipal, India-576104.
Email: sanjiban.c@manipal.edu

***Corresponding Author:** Ravindranath Bilachi Sanganabasappa.
ravindranath.bs@manipal.edu

Supplementary data for the mRNA expression analysis of PCNA in different cell line categories.

Cell line gropus	No. of Cell lines	Avg. Expression (nTPM)
Lymphoma	76	638.9
Leukemia	93	540.7
Bile duct cancer	7	531.8
Bladder cancer	26	456
Colorectal cancer	63	423.9
Skin cancer	62	384.4
Cervical cancer	8	378.4
Bone cancer	21	376.6
Gastric cancer	42	372
Neuroblastoma	17	364.9
Liver cancer	24	362.5
Breast cancer	62	359.4
Myeloma	34	354
Brain cancer	80	340.7
Kidney cancer	35	340.5
Lung cancer	232	336.3
Esophageal cancer	27	335.3
Prostate cancer	8	331.1
Sarcoma	15	328
Pancreatic cancer	46	326.7
Ovarian cancer	59	321.7
Thyroid cancer	11	313
Rhabdoid	14	311.8
Head and neck cancer	38	303.7
Uncategorised	11	299.1
Uterine cancer	29	282.3
Non- cancerous	63	223.6
Adrenocotical cancer	1	213.6
Testis cancer	1	197
Gallbladder cancer	1	188.4

Table S1: Average mRNA levels of PCNA in different cell lines (Source: HPA database)

Supplementary data for the analysis of mapping pan-cancer expression of PCNA in cancer pathways.

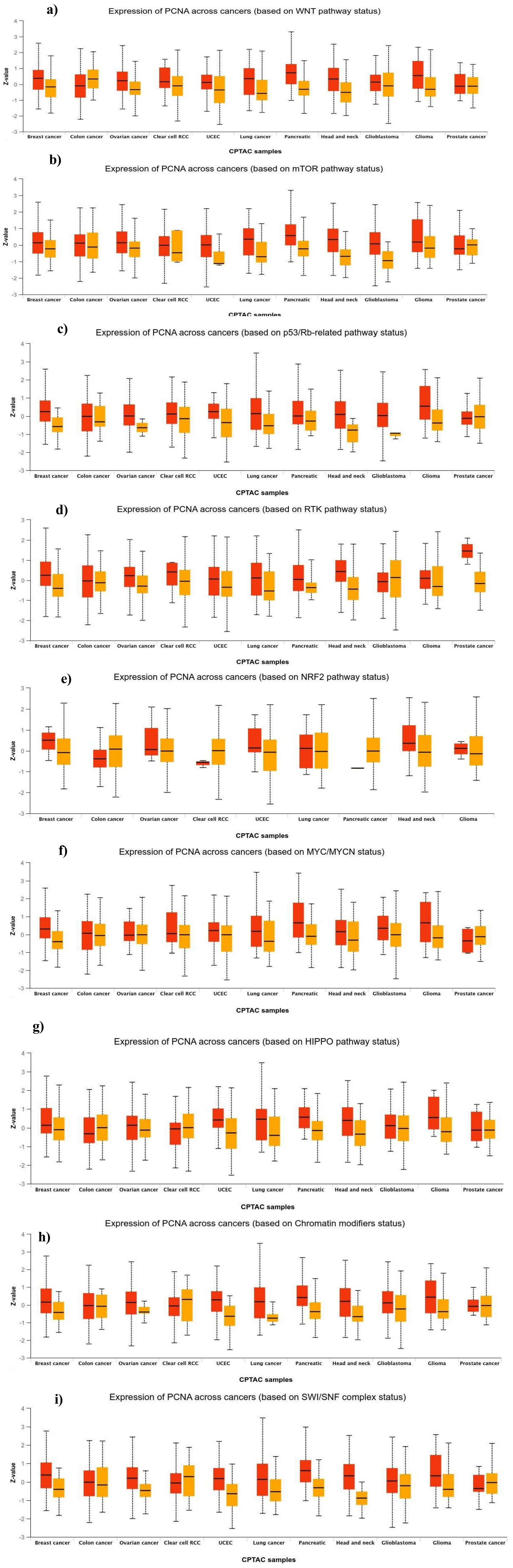

Figure S1 a-i Box plots of PCNA expression level across pan-cancer in altered (red) vs non altered (yellow) in different signalling pathways (Source: UALCAN).

Gene	Pathway	Cancer	Parameters	Altered	Unaltered/Others	Statistical significance	
PCNA	WNT	Breast cancer	MEDIAN	0.376	-0.171	3.41E-03	Significant
PCNA	WNT	Ovarian	MEDIAN	0.233	-0.341	1.99E-02	Significant
PCNA	WNT	UCEC	MEDIAN	0.119	-0.361	1.64E-02	Significant
PCNA	WNT	Lung cancer	MEDIAN	0.35	-0.562	9.97E-04	Significant
PCNA	mTOR	Breast cancer	MEDIAN	0.137	-0.217	2.69E-02	Significant
PCNA	mTOR	Ovarian	MEDIAN	0.141	-0.179	1.75E-02	Significant
PCNA	mTOR	Lung cancer	MEDIAN	0.35	-0.695	9.61E-05	Significant
PCNA	mTOR	Pancreatic cancer	MEDIAN	0.581	-0.225	1.58E-05	Significant
PCNA	mTOR	Head and neck cancer	MEDIAN	0.334	-0.687	3.11E-07	Significant
PCNA	mTOR	Glioblastoma	MEDIAN	0.085	-0.934	4.56E-04	Significant
PCNA	p53/Rb	Breast cancer	MEDIAN	0.252	-0.569	3.13E-08	Significant
PCNA	p53/Rb	Clear Cell RCC	MEDIAN	0.127	-0.13	2.73E-02	Significant
PCNA	p53/Rb	UCEC	MEDIAN	0.252	-0.348	3.13E-03	Significant
PCNA	p53/Rb	Lung cancer	MEDIAN	0.14	-0.536	3.06E-02	Significant
PCNA	RTK	Breast cancer	MEDIAN	0.242	-0.403	2.34E-04	Significant
PCNA	RTK	Head and neck cancer	MEDIAN	0.436	-0.439	3.26E-03	Significant
PCNA	NRF2	Pancreatic cancer	MEDIAN	-0.831	-0.003	<1E-12	Significant
PCNA	Chromatin modifier	Breast cancer	MEDIAN	0.173	-0.418	7.07E-05	Significant
PCNA	Chromatin modifier	UCEC	MEDIAN	0.298	-0.634	8.82E-06	Significant
PCNA	Chromatin modifier	Lung cancer	MEDIAN	0.178	-0.74	1.39E-05	Significant
PCNA	Chromatin modifier	Pancreatic cancer	MEDIAN	0.425	-0.382	1.52E-08	Significant
PCNA	Chromatin modifier	Head and neck cancer	MEDIAN	0.198	-0.655	6.34E-04	Significant
PCNA	Chromatin modifier	Glioblastoma	MEDIAN	0.121	-0.229	3.83E-02	Significant
PCNA	MYC/MYCN	Breast cancer	MEDIAN	0.308	-0.403	4.00E-04	Significant
PCNA	MYC/MYCN	Lung cancer	MEDIAN	0.193	-0.377	3.33E-02	Significant
PCNA	MYC/MYCN	Pancreatic cancer	MEDIAN	0.659	-0.101	6.59E-03	Significant
PCNA	SWI/SNF	Breast cancer	MEDIAN	0.372	-0.394	1.08E-06	Significant
PCNA	SWI/SNF	Ovarian	MEDIAN	0.206	-0.474	4.80E-02	Significant
PCNA	SWI/SNF	UCEC	MEDIAN	0.188	-0.632	1.01E-05	Significant
PCNA	SWI/SNF	Lung cancer	MEDIAN	0.14	-0.536	2.12E-02	Significant
PCNA	SWI/SNF	Pancreatic cancer	MEDIAN	0.612	-0.318	8.67E-10	Significant
PCNA	SWI/SNF	Head and neck cancer	MEDIAN	0.344	-0.869	3.40E-09	Significant
PCNA	HIPPO	UCEC	MEDIAN	0.422	-0.27	8.91E-04	Significant
PCNA	HIPPO	Lung cancer	MEDIAN	0.464	-0.395	1.18E-02	Significant
PCNA	HIPPO	Pancreatic cancer	MEDIAN	0.581	-0.136	1.76E-03	Significant
PCNA	HIPPO	Head and neck cancer	MEDIAN	0.395	-0.327	1.08E-03	Significant
PCNA	WNT	Colon cancer	MEDIAN	-0.1	0.331	1.76E-01	Insignificant
PCNA	WNT	Clear Cell RCC	MEDIAN	0.173	-0.095	1.67E-01	Insignificant
PCNA	WNT	Pancreatic cancer	MEDIAN	0.724	-0.303	NA	Insignificant
PCNA	WNT	Head and neck cancer	MEDIAN	0.344	-0.512	NA	Insignificant
PCNA	WNT	Glioblastoma	MEDIAN	0.141	-0.093	NA	Insignificant
PCNA	WNT	Glioma	MEDIAN	0.557	-0.316	NA	Insignificant
PCNA	WNT	Prostate cancer	MEDIAN	-0.114	-0.128	8.17E-01	Insignificant
PCNA	mTOR	Colon cancer	MEDIAN	0.12	-0.117	8.52E-01	Insignificant
PCNA	mTOR	Clear Cell RCC	MEDIAN	0	-0.468	8.08E-01	Insignificant
PCNA	mTOR	UCEC	MEDIAN	0.008	-1.118	1.89E-01	Insignificant
PCNA	mTOR	Glioma	MEDIAN	0.191	-0.191	NA	Insignificant
PCNA	mTOR	Prostate cancer	MEDIAN	-0.232	0.021	3.38E-01	Insignificant
PCNA	p53/Rb	Colon cancer	MEDIAN	-0.004	-0.309	6.81E-01	Insignificant
PCNA	p53/Rb	Ovarian	MEDIAN	0.002	-0.631	3.79E-01	Insignificant
PCNA	p53/Rb	Pancreatic cancer	MEDIAN	0.018	-0.262	NA	Insignificant
PCNA	p53/Rb	Head and neck cancer	MEDIAN	0.099	-0.778	NA	Insignificant
PCNA	p53/Rb	Glioblastoma	MEDIAN	0.04	-0.935	NA	Insignificant
PCNA	p53/Rb	Glioma	MEDIAN	0.559	-0.387	NA	Insignificant
PCNA	p53/Rb	Prostate cancer	MEDIAN	-0.114	-0.026	9.25E-01	Insignificant
PCNA	RTK	Colon cancer	MEDIAN	-0.031	-0.128	9.79E-01	Insignificant
PCNA	RTK	Ovarian	MEDIAN	0.233	-0.295	1.70E-01	Insignificant
PCNA	RTK	Clear Cell RCC	MEDIAN	0.419	-0.05	3.94E-01	Insignificant
PCNA	RTK	UCEC	MEDIAN	0.073	-0.345	1.64E-01	Insignificant
PCNA	RTK	Lung cancer	MEDIAN	0.113	-0.524	8.40E-02	Insignificant
PCNA	RTK	Pancreatic cancer	MEDIAN	0.041	-0.356	1.44E-01	Insignificant
PCNA	RTK	Glioblastoma	MEDIAN	-0.065	0.141	6.60E-01	Insignificant
PCNA	RTK	Glioma	MEDIAN	0.101	-0.298	NA	Insignificant
PCNA	RTK	Prostate cancer	MEDIAN	1.45	-0.161	2.31E-01	Insignificant
PCNA	NRF2	Breast cancer	MEDIAN	0.508	-0.081	3.62E-01	Insignificant
PCNA	NRF2	Colon cancer	MEDIAN	-0.379	0.079	1.08E-01	Insignificant
PCNA	NRF2	Ovarian	MEDIAN	0.065	-0.007	5.63E-01	Insignificant
PCNA	NRF2	Clear Cell RCC	MEDIAN	-0.557	0.007	6.01E-01	Insignificant
PCNA	NRF2	UCEC	MEDIAN	0.138	-0.066	7.56E-02	Insignificant
PCNA	NRF2	Lung cancer	MEDIAN	0.127	-0.028	8.66E-01	Insignificant
PCNA	NRF2	Head and neck cancer	MEDIAN	0.367	-0.068	5.23E-02	Insignificant
PCNA	NRF2	Glioma	MEDIAN	0.114	-0.146	NA	Insignificant
PCNA	Chromatin modifier	Colon cancer	MEDIAN	-0.031	-0.076	7.10E-01	Insignificant
PCNA	Chromatin modifier	Ovarian	MEDIAN	0.141	-0.39	9.70E-02	Insignificant
PCNA	Chromatin modifier	Clear Cell RCC	MEDIAN	-0.061	0.309	7.17E-01	Insignificant
PCNA	Chromatin modifier	Glioma	MEDIAN	0.447	-0.388	NA	Insignificant
PCNA	Chromatin modifier	Prostate cancer	MEDIAN	-0.081	-0.026	1.00E+00	Insignificant
PCNA	MYC/MYCN	Colon cancer	MEDIAN	0.08	-0.054	8.67E-01	Insignificant
PCNA	MYC/MYCN	Ovarian	MEDIAN	-0.037	-0.002	8.46E-01	Insignificant
PCNA	MYC/MYCN	Clear Cell RCC	MEDIAN	0.061	-0.001	1.38E-01	Insignificant
PCNA	MYC/MYCN	UCEC	MEDIAN	0.229	-0.017	1.91E-01	Insignificant
PCNA	MYC/MYCN	Head and neck cancer	MEDIAN	0.157	-0.318	7.42E-02	Insignificant
PCNA	MYC/MYCN	Glioblastoma	MEDIAN	0.351	-0.005	3.04E-01	Insignificant
PCNA	MYC/MYCN	Glioma	MEDIAN	0.657	-0.182	NA	Insignificant
PCNA	MYC/MYCN	Prostate cancer	MEDIAN	-0.349	-0.114	5.73E-01	Insignificant
PCNA	SWI/SNF	Colon cancer	MEDIAN	-0.008	-0.169	9.12E-01	Insignificant
PCNA	SWI/SNF	Clear Cell RCC	MEDIAN	-0.05	0.286	5.75E-01	Insignificant
PCNA	SWI/SNF	Glioblastoma	MEDIAN	0.057	-0.204	1.97E-01	Insignificant
PCNA	SWI/SNF	Glioma	MEDIAN	0.345	-0.396	NA	Insignificant
PCNA	SWI/SNF	Prostate cancer	MEDIAN	-0.351	-0.026	7.98E-01	Insignificant
PCNA	HIPPO	Breast cancer	MEDIAN	0.137	-0.098	1.18E-01	Insignificant
PCNA	HIPPO	Colon cancer	MEDIAN	-0.32	0.016	4.07E-01	Insignificant
PCNA	HIPPO	Ovarian	MEDIAN	0.136	-0.112	9.82E-01	Insignificant
PCNA	HIPPO	Clear Cell RCC	MEDIAN	-0.05	0.007	4.81E-01	Insignificant
PCNA	HIPPO	Glioblastoma	MEDIAN	0.113	-0.03	6.86E-01	Insignificant
PCNA	HIPPO	Glioma	MEDIAN	0.559	-0.208	NA	Insignificant
PCNA	HIPPO	Prostate cancer	MEDIAN	-0.114	-0.128	7.18E-01	Insignificant

Table S2: Median expression values of PCNA in several cancer in altered and unaltered signalling pathways, retrieved from UALCAN database

Supplementary data for heatmap interaction of selected pathways and enriched mapped genes from STRING database (KEGG, Wiki pathways, Reactome) for gene enrichment analysis of PCNA.

Term ID	Database	Pathway description	Genes
hsa04115	KEGG(STRING)	Activation of NF-kappaB in B cells	CCND1 GADD45G CDK4 CCND2 CDK6 CDK2 GADD45A CCND3 CDKN1A
hsa05200	KEGG(STRING)	ATM signaling pathway	CCND1 MSH2 MSH6 POLK GADD45G CDK4 CCND2 MSH3 CDK6 CDK2 GADD45A CCND3 CDKN1A
hsa04068	KEGG(STRING)	Constitutive Signaling by Ligand-Responsive EGFR Cancer Variants	CCND1 GADD45G CCND2 CDK2 GADD45A CDKN1A
hsa04151	KEGG(STRING)	Defective binding of RB1 mutants to E2F1,(E2F2, E2F3)	CCND1 CDK4 CCND2 CDK6 CDK2 CCND3 CDKN1A
hsa05206	KEGG(STRING)	Downregulation of ERBB2:ERBB3 signaling	CCND1 CCND2 CDK6 DNMT1 CDKN1A
hsa05202	KEGG(STRING)	Downregulation of ERBB4 signaling	POLK GADD45G CCND2 GADD45A CDKN1A
hsa04630	KEGG(STRING)	Downregulation of SMAD2/3:SMAD4 transcriptional activity	CCND1 CCND2 CCND3 CDKN1A
hsa04340	KEGG(STRING)	Downregulation of TGF-beta receptor signaling	CCND1 CCND2
WP2446	Wiki Pathways(STRING)	Extension of Telomeres	CCND1 MSH6 RPA1 CDK4 MCM4 POLD3 CDK6 CDK2 CDT1 MCM7 HLTF POLE PRIM1 DNMT1 CCND3 POLA1 PCNA RFC3 RFC4 CDKN1A RFC5 MCM3
WP4963	Wiki Pathways(STRING)	FLT3 signaling in disease	MSH2 POLK CDK2 GADD45A POLH PCNA CDKN1A
WP1971	Wiki Pathways(STRING)	FoxO signaling pathway	MSH2 MSH6 CDK4 CDK2 CDKN1A
WP4172	Wiki Pathways(STRING)	FOXO-mediated transcription of cell cycle genes	CCND1 CDK4 CCND2 CDK6 CDK2 CCND3 CDKN1A
WP2516	Wiki Pathways(STRING)	H19 action Rb-E2F1 signaling and CDK-Beta-catenin activity	CDK2 GADD45A CDKN1A
WP3969	Wiki Pathways(STRING)	Hedgehog signaling pathway	CCND1 CDK4
WP1742	Wiki Pathways(STRING)	Integrated cancer pathway	GADD45A CDKN1A
WP399	Wiki Pathways(STRING)	JAK-STAT signaling pathway	CCND1 CCND2 CCND3
WP4204	Wiki Pathways(STRING)	JNK (c-Jun kinases) phosphorylation and activation mediated by activated human TAK1	CDK4 CDK6
WP428	Wiki Pathways(STRING)	KEAP1-NFE2L2 pathway	CCND1 CCND2 CCND3
HSA-180786	Reactome (STRING)	MAPK6/MAPK4 signaling	RFC2 RPA1 CHTF18 LIG1 POLD3 CDK2 FEN1 POLD4 PRIM1 POLA1 PCNA RFC3 RFC1 RFC4 RFC5 POLD1 POLD2 PRIM2
HSA-6804756	Reactome (STRING)	MicroRNAs in cancer	RFC2 RPA1 CDK2 RPS27A EXO1 RFC3 RFC4 UBA52 RFC5 UBC UBB
HSA-3700989	Reactome (STRING)	Negative regulation of MAPK pathway	RFC2 MSH2 RPA1 CDK2 RPS27A EXO1 GADD45A PCNA RFC3 RFC4 CDKN1A UBA52 RFC5 UBC UBB
HSA-9661069	Reactome (STRING)	NOD1/2 Signaling Pathway	CCND1 CDK4 CCND2 CDK6 CDK2 CCND3 CDKN1A
HSA-187577	Reactome (STRING)	NOTCH1 Intracellular Domain Regulates Transcription	CCND1 CDK4 CDK2 RPS27A CDKN1A UBA52 UBC UBB
HSA-2559582	Reactome (STRING)	p53 signaling pathway	CDK4 CDK6 CDK2 RPS27A CDKN1A UBA52 UBC UBB
HSA-8848021	Reactome (STRING)	p53 transcriptional gene network	CCND1 CDK4 CDK2 RPS27A UBA52 UBC UBB
HSA-9682385	Reactome (STRING)	Pathways in cancer	RPS27A CDKN1A UBA52 UBC UBB
HSA-1253288	Reactome (STRING)	PI3K-Akt signaling pathway	RPS27A UBA52 UBC UBB
HSA-8849469	Reactome (STRING)	PI3K-Akt signaling pathway	RPS27A UBA52 UBC UBB
HSA-8948747	Reactome (STRING)	PIP3 activates AKT signaling	RPS27A UBA52 UBC UBB
HSA-1358803	Reactome (STRING)	PTK6 Regulates Cell Cycle	RPS27A UBA52 UBC UBB
HSA-8878159	Reactome (STRING)	PTK6 Regulates RTKs and Their Effectors AKT1 and DOK1	CCND1 RPS27A CDKN1A UBA52 UBC UBB
HSA-1236382	Reactome (STRING)	RAS processing	RPS27A UBA52 UBC UBB
HSA-450321	Reactome (STRING)	Regulation of activated PAK-2p34 by proteasome mediated degradation	RPS27A UBA52 UBC UBB
HSA-9648002	Reactome (STRING)	Regulation of PTEN localization	RPS27A UBA52 UBC UBB
HSA-2173788	Reactome (STRING)	Regulation of RAS by GAPs	RPS27A UBA52 UBC UBB
HSA-8849470	Reactome (STRING)	Regulation of TNFR1 signaling	CCND1 CDK4 CDK2
HSA-2173795	Reactome (STRING)	Regulation of TP53 Activity through Phosphorylation	RPS27A UBA52 UBC UBB
HSA-168638	Reactome (STRING)	Retinoblastoma gene in cancer	RPS27A UBA52 UBC UBB
HSA-5687128	Reactome (STRING)	SCF(Skp2)-mediated degradation of p27/p21	RPS27A CCND3 UBA52 UBC UBB
HSA-5675221	Reactome (STRING)	Senescence-Associated Secretory Phenotype (SASP)	RPS27A UBA52 UBC UBB
HSA-5357905	Reactome (STRING)	Signaling by NOTCH	RPS27A UBA52 UBC UBB
HSA-9755511	Reactome (STRING)	Signaling by PTK6	RPS27A CDKN1A UBA52 UBC UBB
HSA-2122947	Reactome (STRING)	SUMO E3 ligases SUMOylate target proteins	RPS27A UBA52 UBC UBB
HSA-211733	Reactome (STRING)	TP53 network	RPS27A UBA52 UBC UBB
HSA-1169091	Reactome (STRING)	Transcriptional misregulation in cancer	RPS27A UBA52 UBC UBB
HSA-5658442	Reactome (STRING)	Transcriptional regulation by RUNX3	RPS27A UBA52 UBC UBB
HSA-157118	Reactome (STRING)	Transcriptional Regulation by TP53	CCND1 RPS27A UBA52 UBC UBB
HSA-1257604	Reactome (STRING)	Tumor suppressor activity of SMARCB1	RPS27A CDKN1A UBA52 UBC UBB
HSA-3108232	Reactome (STRING)	Wnt signaling	RPA1 UHRF2 DNMT1 PCNA
HSA-9617828	Reactome (STRING)	Wnt signaling pathway and pluripotency	GADD45A CDKN1A

Table S3. Enrichment analysis of PCNA and its interactors using the STRING tool, based on selectively chosen pathways from different databases relevant to cancer biology

Supplementary data for ppi network and gene enrichment analysis of PCNA.

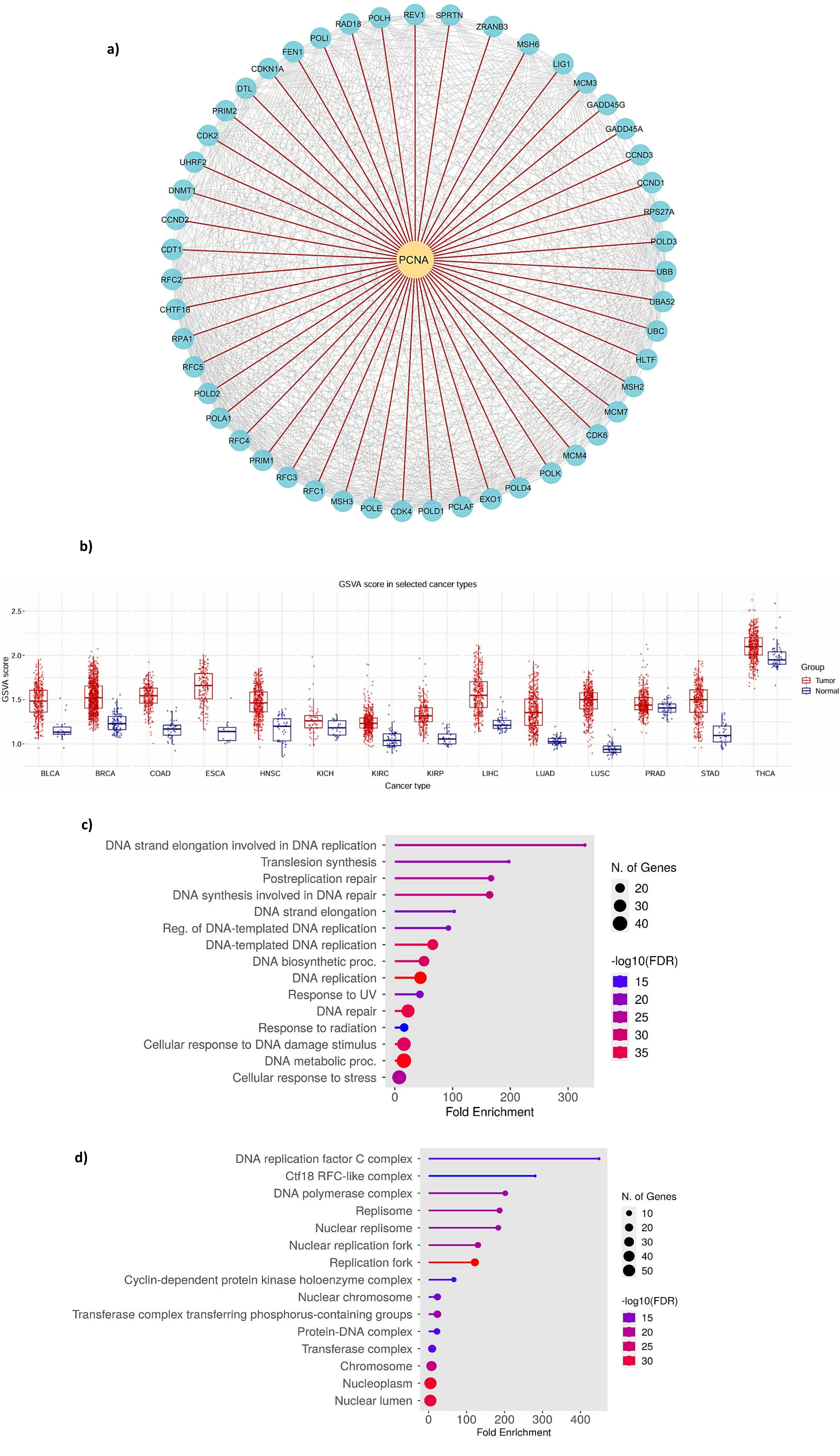

a)
PCNA
REV1
SPRTN
ZRANB3
MSH6
LIG1
MCM3
GADD45G
GADD45A
CCND3
CCND1
RPS27A
POLD3
UBB
UBA52
UBC
HLTF
MSH2
MCM7
CDK6
MCM4
POLK
POLD4
EXO1
PCLAF
POLD1
CDK4
POLE
MSH3
RFC1
RFC3
PRIM1
RFC4
POLA1
POLD2
RFC5
RPA1
CHTF18
RFC2
CDT1
CCND2
DNMT1
UHRF2
CDK2
PRIM2
DTL
CDKN1A
FEN1
POLI
RAD18
POLH
b)
GSVA score in selected cancer types
GSVA score
2.5
2.0
1.5
1.0
BLCA
BRCA
COAD
ESCA
HNSC
KICH
KIRC
KIRP
LIHC
LUAD
LUSC
PRAD
STAD
THCA
Cancer type
Group
Tumor
Normal
c)
DNA strand elongation involved in DNA replication
Translesion synthesis
Postreplication repair
DNA synthesis involved in DNA repair
DNA strand elongation
Reg. of DNA-templated DNA replication
DNA-templated DNA replication
DNA biosynthetic proc.
DNA replication
Response to UV
DNA repair
Response to radiation
Cellular response to DNA damage stimulus
DNA metabolic proc.
Cellular response to stress
0
100
200
300
Fold Enrichment
N. of Genes
20
30
40
-log10(FDR)
15
20
25
30
35
d)
DNA replication factor C complex
Ctf18 RFC-like complex
DNA polymerase complex
Replisome
Nuclear replisome
Nuclear replication fork
Replication fork
Cyclin-dependent protein kinase holoenzyme complex
Nuclear chromosome
Transferase complex transferring phosphorus-containing groups
Protein-DNA complex
Transferase complex
Chromosome
Nucleoplasm
Nuclear lumen
0
100
200
300
400
Fold Enrichment
N. of Genes
10
20
30
40
50
-log10(FDR)
15
20
25
30

e)

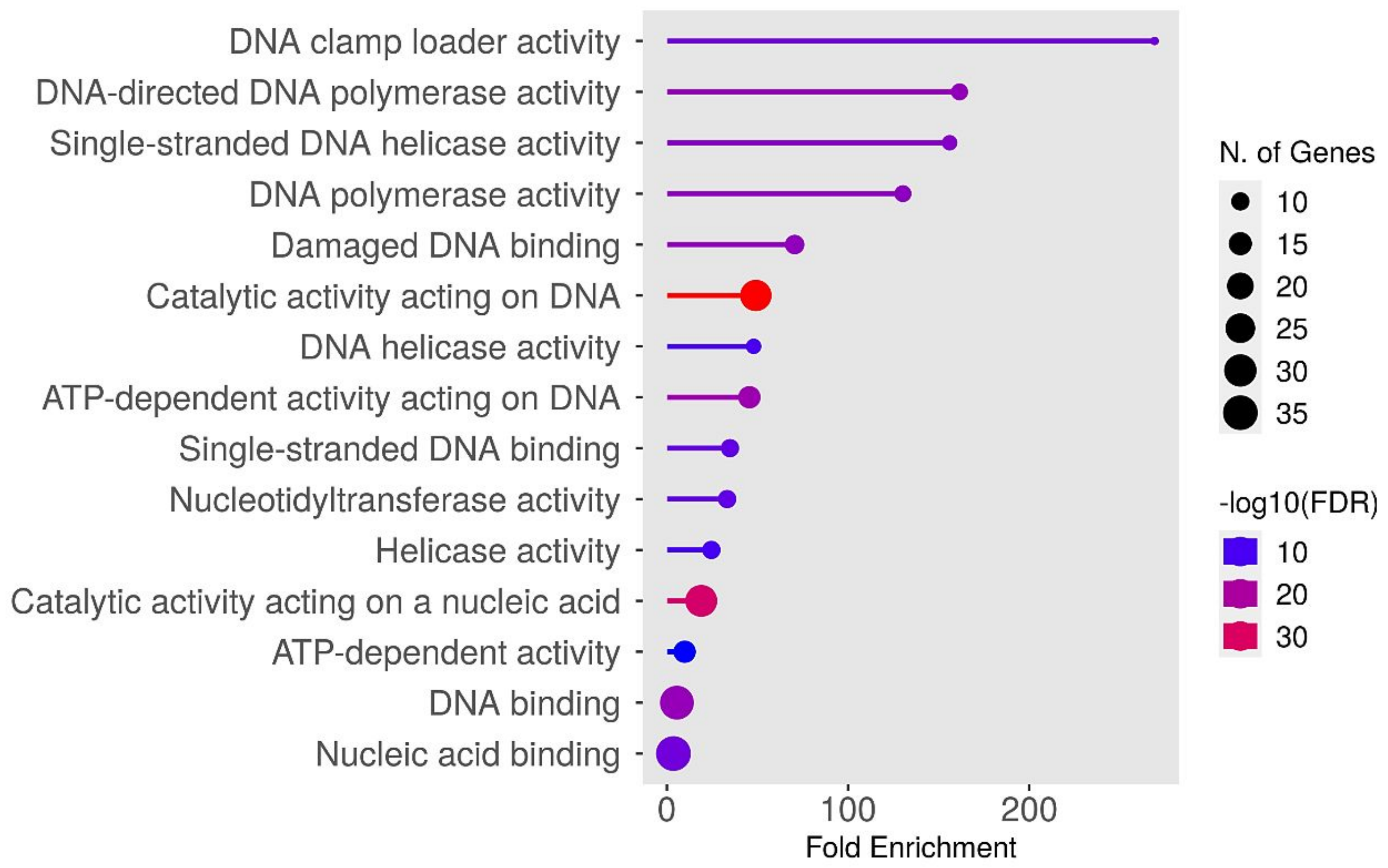

f)

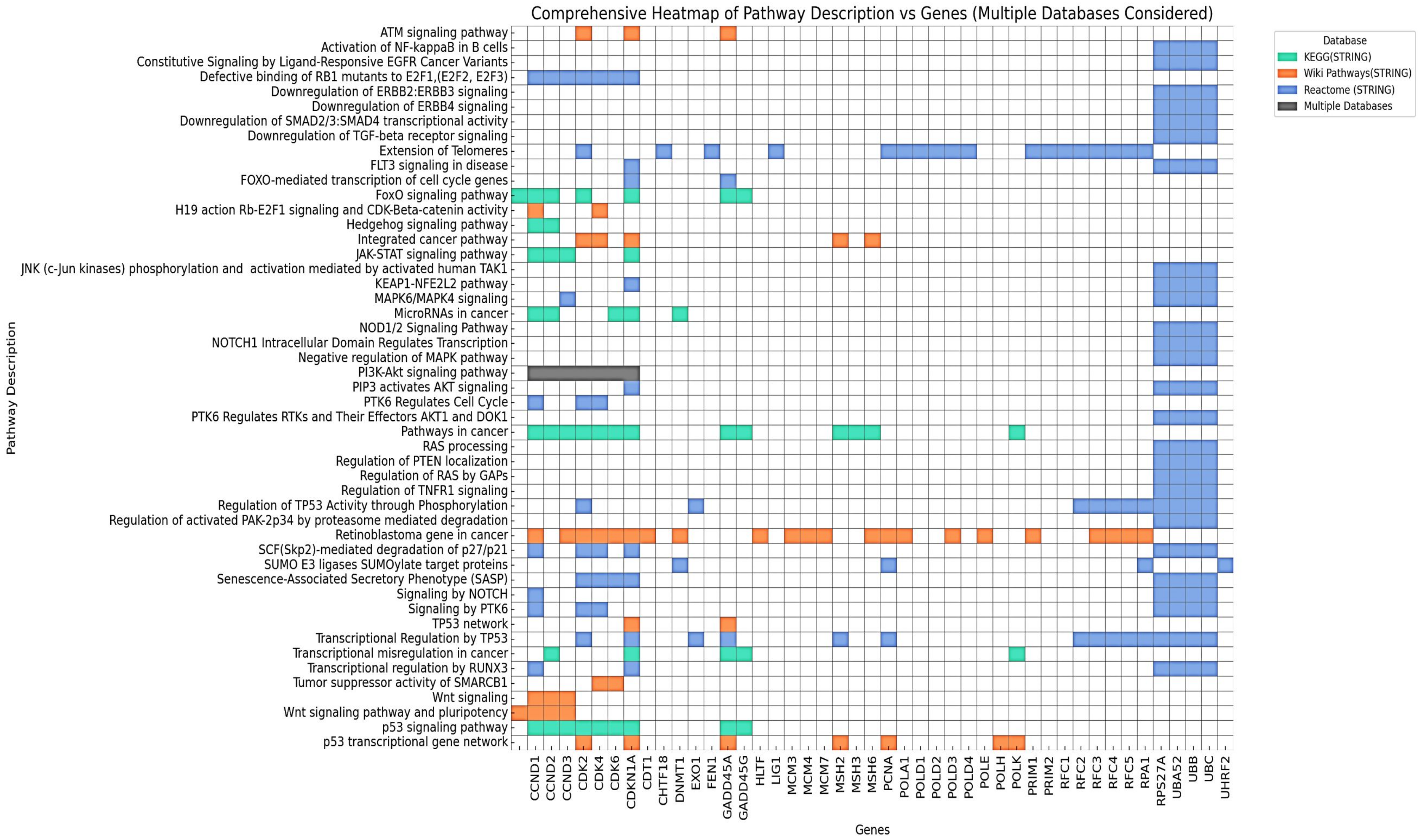

Figure S2 a) Protein-protein interaction network of PCNA with top 50 binding partners. **b)** GSVA score distribution of PCNA and its interactors in selected cancer types from TCGA dataset. **c)** Gene ontology (GO) enrichment for biological process of PCNA and its associated partners. d) GO enrichment for cellular localisation. **e)** GO enrichment for molecular function. **f)** Heatmap interaction of selected pathways and enriched mapped genes .
